# Evolution of Cancer Metastases via Lineage Trans-Differentiation

**DOI:** 10.34133/research.1144

**Published:** 2026-02-19

**Authors:** Yu Xiao, Wan Jin, Fangjin Chen, Kaiyu Qian, Lingao Ju, Yi Zhang

**Affiliations:** ^1^ Department of Biological Repositories, Human Genetic Resources Preservation Center of Hubei Province, Laboratory of Precision Medicine, Zhongnan Hospital of Wuhan University, Wuhan, China.; ^2^ Institute for Genetics and Molecular Medicine, Chinese Institutes for Medical Research, Beijing, China.; ^3^ High Performance Computing Center, Peking-Tsinghua College of Life Sciences, Peking University, Beijing, China.; ^4^ Department of Urology, Hubei Key Laboratory of Urological Diseases, Zhongnan Hospital of Wuhan University, Wuhan, China.; ^5^ Medical Research Institute, Frontier Science Center for Immunology and Metabolism, Wuhan University, Wuhan, China.; ^6^ Wuhan Research Center for Infectious Diseases and Cancer, Chinese Academy of Medical Sciences, Wuhan, China.; ^7^ School of Basic Medical Sciences, Capital Medical University, Beijing, China.

## Abstract

Most cancer metastases exhibit mutational profiles similar to those of the primary tumor. However, the nongenetic mechanisms driving metastasis remain poorly understood. Here, we show that lineage trans-differentiation is a hallmark of cancer metastasis. Bioinformatic tools capable of reconstructing cancer phenotypic evolutionary trajectories at single-cell resolution were developed, revealing a progressive loss of transcriptional and epigenomic lineage fidelity in cancer cells as they evolve toward metastasis. During premetastatic evolution, cells undergo de-differentiation into a fetal-like state. The mis-expression of alternative-lineage gene programs during re-differentiation from this fetal-like state leads to the formation of trans-differentiated metastatic cells. This trans-differentiation, rather than fetal-like transcription, constitutes a key feature of metastasis in both humans and mice. In clinical samples, trans-differentiation correlates with histopathological grade, metastatic potential, and patient survival. Additionally, trans-differentiation is associated with gain of oncogenic mitogen-activated protein kinase (MAPK) signaling and can be reversed through MAPK inhibition. These findings offer a detailed account of metastatic cancer evolution driven by epigenetic reprogramming while also uncovering the molecular mechanisms underlying this process.

## Introduction

Metastatic disease is the leading cause of cancer-related mortality [1]. Extensive molecular genetic profiling has failed to identify consistent alterations in metastatic tumors compared to their primary counterparts [2–9]. Generally, metastases share the same spectrum of driver mutations as the primary tumor, suggesting that new mutations are passive “passengers” that are not required for metastatic dissemination. Although certain metastasis-specific chromosomal aberrations (such as karyotypic or copy number changes) have been observed in some cancers, these are not universally present, and their functional relevance remains unclear [10,11]. In most cases, the genetic mutations in primary tumors appear to facilitate metastatic outgrowth, but do not account for why only a minority of cancer cells in the primary tumor successfully establish distant metastases.

Transcriptional and epigenetic profiling has revealed that metastatic cancers often exhibit substantial intratumoral heterogeneity (ITH), which cannot be fully explained by additional mutations [12–15]. This observation has led to the hypothesis that metastatic potential is associated with a highly plastic subpopulation of cells—often termed “cancer stem cells” or “high plasticity progenitor cells”—which adopt progenitor-like, developmental programs [16–18] that resemble a “fetal-like” state. In fact, many aggressive tumors display gene expression signatures indicative of early embryonic or “fetal-like” cell states, including precursor-, germline-, and placental-like transcriptional programs [19–24]. However, reverting tumor cells to an embryonic state alone is insufficient for malignant progression, as demonstrated by the benign nature of teratomas.

Metastatic tumors are commonly associated with features of epithelial–mesenchymal transition (EMT), an embryonic program that transforms epithelial cells into a mesenchymal phenotype, leading to the loss of apical–basal polarity and enhanced cellular motility and invasiveness [25–27]. Although EMT is often regarded as a phase-shift-like event during tumor progression, transcriptional profiling has revealed that EMT represents a combination of distinct molecular states. Single-cell analyses have identified a hybrid or partial EMT state as permissive for metastasis [28–32], while cells undergoing full EMT tend to enter a quiescent, dormant state, limiting their metastatic potential [33]. In clinical settings, tumors with high Ki67 expression are typically considered more aggressive. Additionally, single-cell DNA sequencing has shown that highly proliferative cancer subclones are more efficient at forming metastases. Consequently, the EMT phenotype does not always correlate with metastatic potential. Moreover, a causal link between EMT and metastasis has yet to be experimentally established in vivo. Therefore, the role of EMT and fetal-like transcriptional programs in generating ITH and producing metastasis-competent clones remains unclear.

This study provides evidence that metastasis evolves through lineage trans-differentiation—an alteration in cell phenotype across developmental lineage boundaries. By tracking the transcriptional and epigenetic profiles of cancer cells at single-cell resolution, this study demonstrates that tumor cells progressively lose their original lineage identity. They first undergo de-differentiation into a fetal-like, EMT-active state and subsequently re-differentiate into alternative-lineage fates, producing clones that are primed for metastasis. This trans-differentiation process, rather than the fetal-like progenitor state itself, is a defining feature of cancer metastases in both mouse models and human cancers. This process is driven by heightened mitogen-activated protein kinase (MAPK) signaling activity and can be reversed with MAPK inhibition. These findings, consistent with multiple human clinical trial results, suggest that targeted inhibition of lineage trans-differentiation could be a promising strategy to prevent cancer metastasis.

## Results

### Trans-differentiation of mouse lung cancer during metastasis evolution

**Fig. 1.**
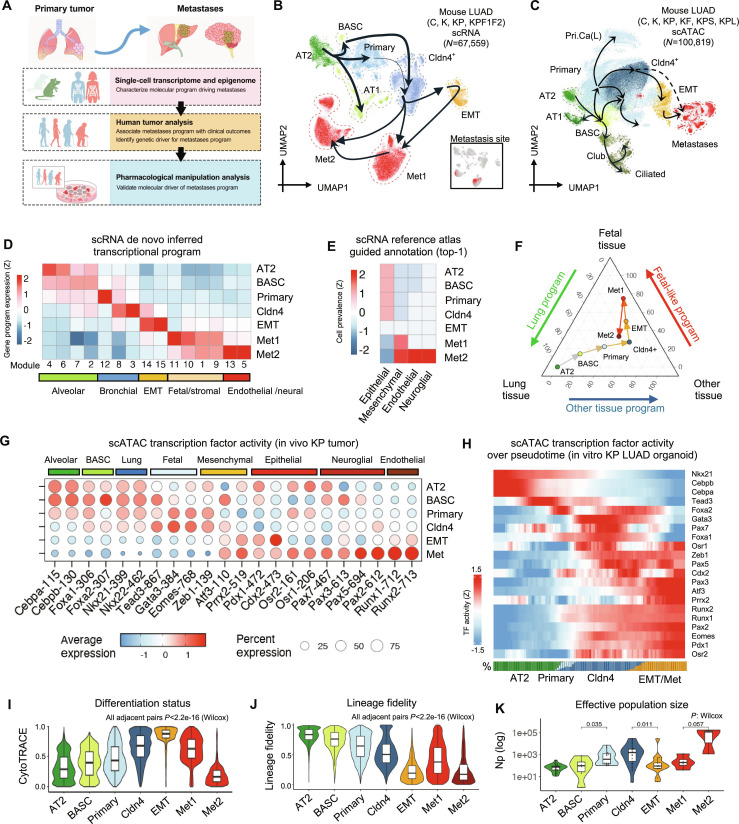
Trans-differentiation of mouse lung cancer during evolution toward metastases. (A) Study scheme. Metastasis-associated transcriptional programs are identified through comparative analysis of single-cell sequencing data from primary cancer and metastases. Human tumor samples were analyzed to validate the clinical phenotypes linked to the identified programs and to identify the genetic variations driving their expression. Finally, pharmacological perturbation datasets were analyzed to validate the driver events for metastasis programs. (B) UMAP of integrated mouse genetically induced LUAD scRNA cells (*N* = 67,559). Inset: Distribution of metastatic cells on the UMAP. (C) UMAP of integrated mouse genetically induced LUAD scATAC cells (*N* = 100,819). (D) Relative expression of de novo inferred transcription modules in mouse LUAD scRNA cells. (E) Distribution of the most similar normal cell type from the Mouse Cell Atlas to mouse LUAD scRNA cells. (F) Median expression of lung-like, fetal-like, and alternative-lineage (other tissue) gene programs in each class of mouse LUAD scRNA cells. (G) TF activities of lung-like, fetal-like, and alternative-lineage (other tissue) master TFs in each class of mouse LUAD scATAC cells. (H) TF activities of lung-like, fetal-like, and alternative-lineage (other tissue) master TFs in mouse LUAD organoid scATAC cells along evolution pseudotime. Bottom: Prevalence of each class of cancer cells at given pseudotime. (I) Stemness of mouse LUAD scRNA cells. (J) Lineage fidelity of mouse LUAD scRNA cells. (K) Total size of progenitor cell estimation for each single-cell clusters. *P* value: Wilcox test. Cell clusters: AT2, type 2 alveolar cells; AT1, type 1 alveolar cells; trAT2, stressed AT2 cells; BASC, bronchioalveolar stem cells; Primary, primary cancer cells; Cldn4^+^, Cldn4^+^ cancer cells; EMT, epithelial–mesenchymal transformation cells; Met1/2, metastatic cluster; Pri.Ca(L), LKB1 mutated primary cancer cells; Club, club cells; Ciliated, ciliated cells of the bronchus. Genotypes: C, wild-type; K, Kras^CA^; KP, Kras^CA^/Trp53^KO^; KF, Kras^CA^/Trp53^hypermorph^; KPF1F2, Kras^CA^/Trp53^KO^/Foxa1^KO^/Foxa2^KO^; KPS, Kras^CA^/Trp53^KO^/Smarca1^KO^; KPL, Kras^CA^/Trp53^KO^/Lkb1^KO^.

To investigate the evolution of metastatic tumors in detail (Fig. 1A), a well-characterized, genetically defined lung adenocarcinoma (LUAD) mouse model was analyzed. In this model, lung alveolar type 2 (AT2) cells were transformed into adenocarcinoma through cell-type-specific Cre-induced overexpression of a constitutively active form of oncogene *Kras* (*Kras^CA^*) [34], which serves as the central signaling hub in the receptor tyrosine kinase (RTK)–Ras–Raf–MAPK pathway (referred to here as the MAPK pathway) [35] (Fig. S1). The MAPK pathway is one of the most frequently altered molecular pathways in human cancers [36]. In humans, the majority of LUADs are driven by oncogenic mutations that hyperactivate MAPK signaling [37–40].

Multiple published single-cell RNA sequencing (scRNA) [41–43] and single-cell assay for transposase-accessible chromatin using sequencing (ATAC-seq, scATAC) datasets [44–48] were integrated to analyze the in vivo evolution of mouse LUAD samples, spanning early to late metastatic stages (Fig. 1B and C). Single cells in the scRNA dataset were classified based on canonical marker gene expression [49] into normal AT2 (AT2: *Lamp3^+^/Sftpb^+^/Nkx2-1^+^*), stressed AT2 (trAT2: *Ifit3^+^/Irf7^+^*), normal AT1 (AT1: *Ager2^+^/Hopx^+^/Nkx2-1^+^*), bronchioalveolar stem cell-like cells (BASC: *Sox2^+^/Cyp2f2^+^/Scgb1a1^+^/Krt8^+^*), primary cancer (Primary: *Gdf15^+^/Cd24^+^/Apoe^+^*), Cldn4^+^ hyperplastic cancer cells (Cldn4^+^: *Cldn4^+^/Tm4sf1^+^/Slc4a11^+^*) [41], EMT cells (EMT: *Vim^+^*) [26], and 2 clusters of cells that could be found at metastatic sites [43] (Met1: *Id3^+^/Gpx3^+^/Sox9^+^/Lgals1^+^*; Met2: *Id3^+^/Gpx3^+^/Bmp2^+^/Zeb2^+^/Runx2^+^*) (Fig. 1B and C and Fig. S2A). The scATAC dataset similarly identified AT2, AT1, BASC, primary cancer, Cldn4^+^, and EMT cells (Fig. 1C), with metastatic cells forming a distinct cluster. Nontumorous ciliated and club cells were also present in the scATAC dataset. Furthermore, a unique cluster of primary cancer cells, found only in KPL (KP, *Lkb1*-deficient) tumors, was identified in the scATAC dataset, distinguishing it from KP (*Kras^CA^, Trp53^KO^*) or KPS (KP, *Smarca4*-deficient) models.

Genetic lineage tracing of cells from the scRNA dataset [43] revealed a stepwise evolutionary trajectory originating from AT2 cells. AT2 cells first transition to BASC and primary cancer cells, which then give rise to Cldn4^+^ cells that coexpress airway epithelial progenitor (AEP) markers such as *Tm4sf1* and *Cd24a*. Cldn4^+^ cells then evolve into EMT cells, which serve as precursors for metastatic (Met1/Met2) cells (Fig. S2B). This evolutionary trajectory was further validated by single-cell replication age estimation [50] in a cell-composition balanced scATAC dataset [46] (Fig. S3A and B). The age of cancer cells progressively increases from AT2-like cells to primary cancer cells, Cldn4^+^ cells, followed by EMT and metastatic cells (Fig. S3C). Additionally, a phylogenetic analysis of cell clusters based on chromatin accessibility at clock-like loci revealed a trajectory from AT2 through Cldn4^+^ and EMT cells to metastatic cells (Fig. S3D). Given these results and the observation that some single-cell clusters are not consistently detected across all analyzed datasets, the primary evolutionary trajectory AT2–BASC–Cldn4^+^–EMT–Met was characterized, with other side branches reserved for future studies.

As previously reported [51–54], *Kras^CA^* induces the transformation of AT2 cells toward BASC (Fig. S4A). However, progression to cancer from BASC requires the loss of *Trp53* and functional *Foxa1/Foxa2* activity (Fig. S4A). Following *Trp53* loss, transformation from primary adenocarcinoma to Cldn4^+^, EMT, and metastatic cells occurs gradually over time (Fig. S4B). Consistent with prior studies [1,12–14], it was observed that nongenetic transcriptional reprogramming continues autonomously without the need for additional genetic alterations, suggesting that the evolution may be driven by epigenomic mechanisms.

Next, transcriptional programs associated with metastasis evolution were systematically explored. From *Kras^CA^*, *Trp53^KO^* cancer cells from primary and metastatic tumors (Fig. S5A), 15 unique transcriptional programs (“modules”) [55] were algorithmically identified and manually annotated based on their biological characteristics (Fig. S5B). As expected, lung-like modules (AT1, AT2, Goblet, and AEP) gradually decreased during cancer evolution [56–58], while EMT-associated modules (Early EMT, pEMT, Late EMT) increased over time (Fig. 1D and Fig. S5C) [30,59–64]. Expression of the fetal-like transcriptional program [19–21,24,65–68] was elevated in early metastatic cells (Met1), while alternative-lineage modules (neuro-glial, stromal, and endothelial lineages) [69–72] were particularly increased in late metastatic cells (Met2) (Fig. 1D and Fig. S5C). To validate this analysis, a similarity search was performed comparing cancer cells with normal cells from the Mouse Cell Atlas [73]. Early-stage cancer cells exhibited the highest similarity to normal epithelial cells, whereas metastatic cancer cells showed reduced similarity to epithelial cells and increased similarity to mesenchymal, endothelial, or ectoderm-derived neuron or glia (neuroglial) cells (Fig. 1E). Thus, metastatic cancer cells are transcriptionally reprogrammed to express genes associated with alternative lineages.

In metastatic cells, the increased expression of genes with alternative-lineage specificity could result from either the loss of defined lineage specificity (de-differentiation) or the gain of alternative-lineage specificity (trans-differentiation). Furthermore, trans-differentiation could occur either directly from the original lineage or through re-differentiation from a de-differentiated intermediate state. To distinguish between these possibilities, single-cell gene expression profiles were decomposed into lung-like, fetal-like, and alternative-lineage-like components (Fig. S6A and B). During the evolution toward metastatic cancer, cells first transition from a lung-like state to a fetal-like state and then re-differentiate to express alternative-lineage genes (Fig. 1F and Fig. S6A and B). This result was further validated through de novo inferred transcription module expression in single cells (Fig. S6C). Similarly, analysis of transcription factor (TF) activity in in vivo Kras^CA^; Trp53^KO^ (KP) mouse cancer cells and in vitro KP cancer organoid scATAC datasets [74] showed a decrease in the activity of lung-lineage TFs, such as *Nkx2-1*, *Cebpa*, and *Foxa2*, from AT2 cells, followed by the induction of fetal-lineage TFs like *Gata3*, *Tead3*, and *Eomes* in primary cancer and Cldn4^+^ cells. Alternative-lineage TFs were primarily active in post-EMT metastatic cells (Fig. 1G and H and Fig. S7). In line with this analysis, stemness estimation (cell differentiation state) using cancer cell RNA expression [75] revealed gradual de-differentiation from AT2 cells to EMT cells, followed by re-differentiation from EMT cells to metastatic Met1/Met2 cells (Fig. 1I).

A quantitative metric of single-cell lineage fidelity was introduced (Materials and Methods), defined as the disparity between the highest and remaining expression similarity scores obtained by correlating a single-cell expression profile against a reference normal cell atlas. A large disparity indicates that a cell uniquely aligns with a single lineage, while a small disparity, reflecting comparable similarity across multiple reference types, signifies low lineage fidelity. It was found that cancer cells progressively lose lineage fidelity during metastasis evolution (Fig. 1J).

To validate the decrease in single-cell lineage fidelity during cancer cell evolution, a method was developed using maximum likelihood estimation to compute the probability of a single cell adopting the fate of any reference normal cell in a panel. This was used to derive lineage confusion for each single cell, defined as the total transcriptional distance between reference normal cells most likely to match it (Materials and Methods). The lineage confusion of genetically traced cancer clones [43] along their phylogenetic tree was then tracked (Fig. S8A and B). Compared to normal-like AT2, AT1, and BASC cells, cancer cells were significantly more likely to show increased lineage confusion than their progenitors (Fig. S8C). Furthermore, metastatic cells exhibited increased lineage confusion compared to EMT cells. In metastatic Met2 cells, more than half of the cells showed increased lineage confusion. Collectively, these data suggest that trans-differentiated cancer cells in metastases emerge through de-differentiation followed by re-differentiation.

Early-stage cancers are typically dormant, persisting for many years before clinical detection [76], with metastatic cancer developing many years after initial onset. Several studies have proposed that the evolution of metastatic tumors occurs in a punctuated manner [8,43,77–84]. However, our observations suggest a smooth, continuous transition from the ancestral normal cell to the metastatic cell (Fig. 1J). One possible explanation for this discrepancy is that only a fraction of cancer cells undergo metastatic transformation [84,85]. To test this hypothesis, genetically traced cancer cells [43] were used to compute the total size of progenitor cells (Np, “number of progenitors”) for each cell type cluster [86], revealing a population bottleneck between premetastatic and metastatic cancer stages: Np gradually increases from AT2 to the Cldn4^+^ stage, decreases significantly at the EMT stage, and then increases again after metastatic evolution (Fig. 1K). This suggests that only a subset of premetastatic Cldn4^+^ cells are transformed into EMT cells and beyond.

Analysis of subclusters within the Cldn4^+^ population revealed that these cells exhibit either lung-like chromatin accessibility profiles or profiles resembling those of alternative-lineage cells (Fig. S9A and C). Interestingly, single-cell replicational age analysis shows that a specific Cldn4^+^ subcluster (C5), which does not resemble either lung-like or alternative-like profiles, has the lowest replicational age (Fig. S9B and D). Clusters with high replicational age tend to show lung-like or alternative-lineage-like features, suggesting that Cldn4^+^ cells from the early C5 subcluster could stochastically evolve toward either a lung-like or alternative-like fate (Fig. S9D). Notably, the Cldn4^+^ subcluster with maximal similarity to EMT and metastatic cells comprises only approximately 8% of all Cldn4^+^ cells (Fig. S9D). This proportion is close to the ratio of EMT to Cldn4^+^ progenitor cells inferred from genetic lineage tracing [median Np: 91 (EMT, *N* = 13), 1,553 (Cldn4^+^, *N* = 19), ratio = 5.86%; Fig. 1K and Table S1], supporting the idea that the population bottleneck from Cldn4^+^ to EMT cells is driven by the stochastic nature of epigenomic evolution. Collectively, these findings suggest that only the most de-differentiated Cldn4^+^ cells can undergo EMT, with re-differentiation occurring only after EMT, when global chromatin accessibility is significantly increased [87] (Fig. S10), allowing alternative-lineage TFs to function at full capacity (Fig. 1G and H and Fig. S7).

### Trans-differentiation in human lung cancer during metastasis evolution

**Fig. 2.**
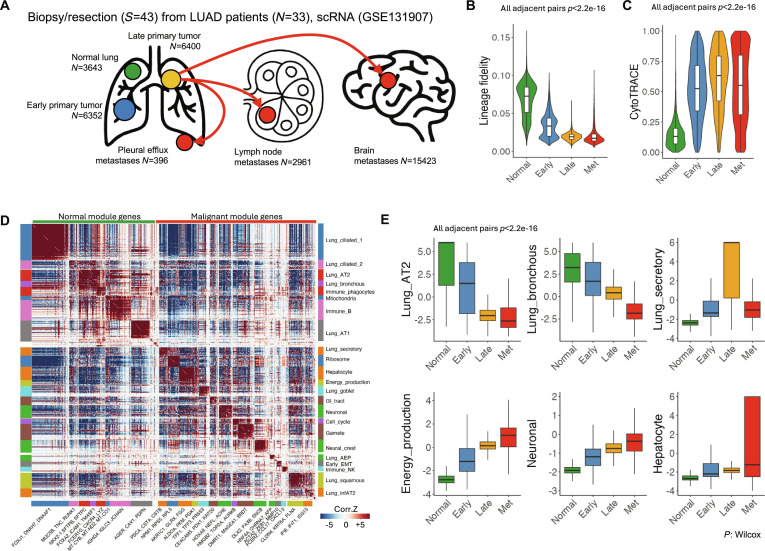
Trans-differentiation of human lung cancer during evolution toward metastases. (A) Study scheme. Normal lung tissue (Normal), early-stage LUAD resection sample (Early), advanced-stage LUAD biopsy (Late), and metastases (Met) samples underwent scRNA. (B) Lineage fidelity of epithelial cells grouped by their stage. (C) Stemness (CytoTRACE score) of epithelial cells. (D) Cross-correlation revealing de novo identified gene coexpression modules in human LUAD single cells. (E) Expression of identified modules across single cells from different stages. Modules specific for terminally differentiated lung cells (AT1, bronchus), intermediate potential lung cells (secretory), energy production, neuronal-like, and hepatocyte-like cells are shown. *P* value: Wilcoxon test.

To assess whether the findings in the mouse model are applicable to human cancer, a re-analysis of an scRNA-seq dataset from patients with LUAD [88] was conducted, covering samples from early to advanced-stage primary tumors and metastases in pleural effusion (PE), lymph nodes (LNs), and brain (Fig. 2A). Similar to the mouse LUAD model, human LUAD cells exhibited a gradual loss of lineage fidelity during their progression (Fig. 2B). Within the primary tumor, a cluster of malignant cells associated with poor clinical outcomes (tS2) [88] demonstrated significantly lower lineage fidelity compared to other malignant cell groups (tS1 and tS3) (Fig. S11A). An alternative validation method, using softmax probability derived from a supervised principal components analysis (PCA)-based cell type annotation approach [89], showed a similar trend of decreasing lineage fidelity with advancing malignancy (Fig. S11B). Concurrently, single-cell stemness progressively increased from normal cells to early and advanced-stage cancer cells, and decreased in metastatic cancer cells, reflecting the trends observed in the mouse model (Fig. 2C and Fig. S11C).

The loss of lineage fidelity could arise from either a reduction in the original cell’s transcriptional program, an increase in the transcriptional program of alternative-lineage cells, or a combination of both. Hotspot [55] gene coexpression modules from these human LUAD cells were segregated into “normal” and “malignant” clusters (Fig. 2D). During LUAD progression, transcription programs associated with the ancestral normal cell (AT2, bronchus) were progressively reduced, while expression of other cell-type-specific transcription programs increased (Fig. 2E and Fig. S12). Thus, at the single-cell level, the reduction in lineage fidelity results from both the loss of original lineage genes and the gain of alternative-lineage gene expression.

A cell with reduced lineage fidelity can adopt either the fate of a promiscuous cell or that of an alternative lineage. Using the maximum likelihood estimation method, it was found that most (97.8% to 98.9%) cancer cells could be unequivocally classified, with >95% probability, as a single terminally differentiated cell type, closely resembling normal cells (98.6% to 99.8%) (Fig. S13A). However, as cancer progresses, the transcriptional profile of cancer cells increasingly deviates from its ancestral cell type (Fig. S13B). In rare instances, cancer cells could be mapped to multiple terminal cell types, with their fate map distribution broadening as cancer evolves, leading to lineage confusion (Fig. S13C and D). These rare multi-mapping cells likely represent an intermediate stage of lineage trans-differentiation.

As trans-differentiation advances, the cancer cell may either adopt the transcriptional profile of an alternative cell within its developmental branch or cross boundaries to resemble cells from other developmental branches, such as cells from different organs. The organ-origin of the best-correlated reference normal cells for these LUAD cells was estimated, and the first hypothesis was rejected: as cancer cells evolve, they gradually transition out of the lung lineage, adopting developmentally distant lineages from other organs (Fig. S14).

### Trans-differentiation is a hallmark of human metastatic cancers

**Fig. 3.**
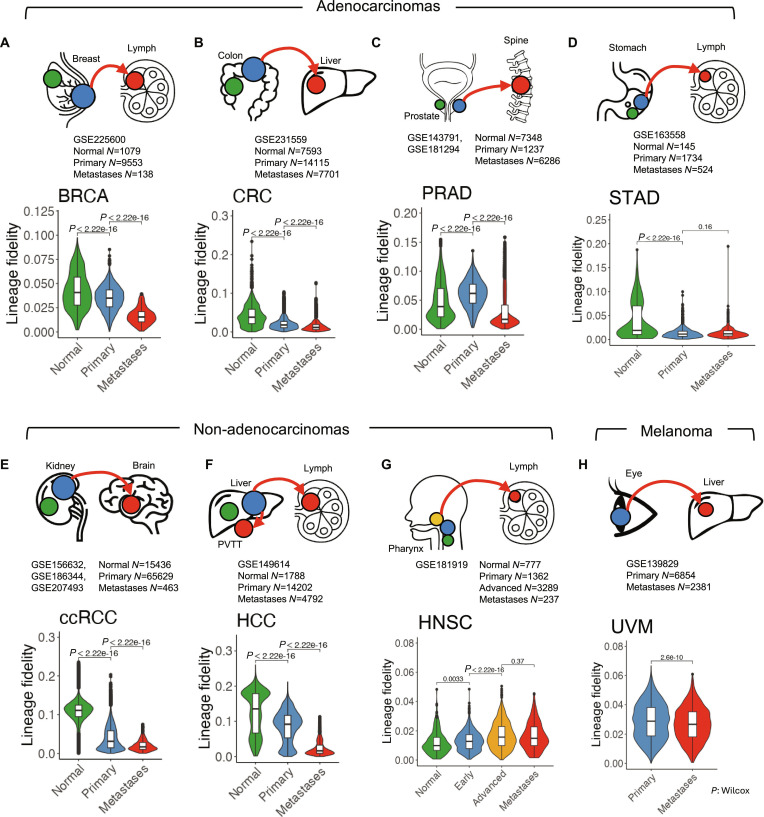
Trans-differentiation is a hallmark of human metastatic cancers. Lineage fidelity scores of normal, primary cancer, and metastatic cells in (A) breast cancer (BRCA), (B) colorectal cancer (CRC), (C) prostate adenocarcinoma (PRAD), (D) stomach adenocarcinoma (STAD), (E) clear cell renal carcinoma (ccRCC), (F) hepatocarcinoma (HCC), (G) head-and-neck squamous carcinoma (HNSC), and (H) uveal melanoma (UVM). For HNSC, primary cancer samples are staged into early and late stages (copresent with metastases). For UVM, no normal cells were available for analysis. *P* value: Wilcoxon test.

Lineage trans-differentiation was identified during the evolution of both mouse and human LUAD toward metastasis, with multiple measures used to distinguish it from other potential confounding events (Fig. S15). However, it remains unclear whether this phenomenon is specific to LUAD or if it can be generalized to other cancer types. To explore this, scRNA-seq data from primary and metastatic human tumor tissues were analyzed. Primary and metastatic data were obtained from the National Center for Biotechnology Information (NCBI) Gene Expression Omnibus (GEO) for 8 tumor types, including 4 adenocarcinomas [breast cancer (BRCA) [90], colorectal cancer (CRC) [91], prostate adenocarcinoma (PRAD) [92,93], and stomach adenocarcinoma (STAD) [94]], 3 non-adenocarcinomas [clear cell renal carcinoma (ccRCC) [95–97], hepatocarcinoma (HCC) [98], and head-and-neck squamous cancer (HNSC) [99]], and 1 melanoma type [uveal melanoma (UVM) [100]]. In all adenocarcinomas and non-adenocarcinomas, except for HNSCs, metastatic cancer cells exhibited a decrease in lineage fidelity compared to primary cells (Fig. 3A to F). Additionally, the stemness of metastatic cancer cells was reduced relative to primary cancer cells (Fig. S16). In HNSC and UVM, although lineage fidelity in metastatic cells did not decrease as markedly as in other tumor types (Fig. 3G and H), the stemness of metastatic cells was still significantly lower (Fig. S16). Therefore, evidence of trans-differentiation in metastatic cells was found across all the tumor types analyzed.

### Epigenomic trans-differentiation in human metastatic cancer

**Fig. 4.**
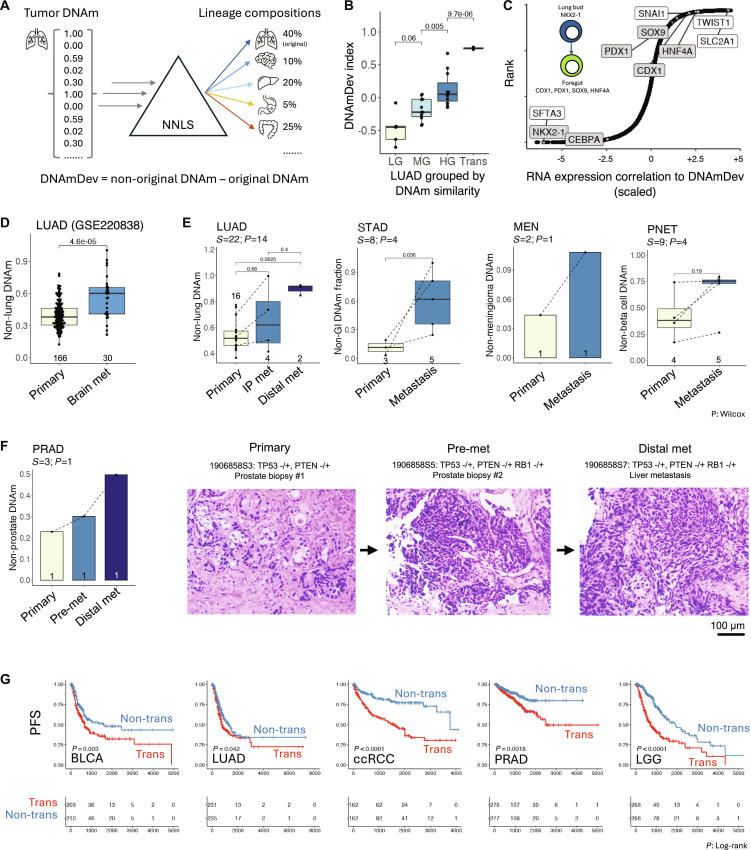
Lineage trans-differentiation in human cancer. (A) Schematic of the process to compute the DNAm-deviation (DNAmDev) index: DNAm values of lineage-specific methylation sites are decomposed using an NNLS method to infer the lineage compositions (weights) of the tumor. DNAmDev is calculated as the difference between the non-original epithelial tissue weight and the original tissue weight. (B) DNAmDev of the Challenge-Lung cohort samples. Samples were grouped by their DNAm similarity to normal tissue references into LG (low-grade), MG (mid-grade), HG (high-grade), and Trans (transformed) classes. (C) Correlation of DNAmDev index with RNA expression of each gene in the TCGA LUAD cohort. Inset: Schematic showing TFs of lung cells and primitive foregut progenitor cells. *P* value: Wilcoxon test. (D) DNAmDev index of primary LUAD and brain metastases in the external cohort [103]. (E) DNAmDev index of in-house cohort primary cancers and metastases. Lines connect samples from the same patient. S, samples; P, patients; STAD, stomach adenocarcinoma; LUAD, lung adenocarcinoma; PNET, pancreatic neuroendocrine tumor; MEN, meningioma. (F) Left: DNAmDev index of samples from a patient with prostate adenocarcinoma (PRAD). Right: Corresponding histological slides from primary, premetastasis, and liver metastasis sites. Scale bar, 100 μm. (G) Progression-free survival (PFS) of TCGA patients with trans-differentiated tumors (DNAmDev > median value) or non-trans-differentiated tumors (DNAmDev ≤ median value). BLCA, bladder cancer; LUAD, lung adenocarcinoma; KIRC, kidney clear cell carcinoma; PRAD, prostate adenocarcinoma; LGG, low-grade glioma. *P* value: log-rank test (for survival) and Wilcoxon test (for 2-group comparison).

In the mouse model, lineage trans-differentiation at the transcriptional level is accompanied by epigenomic reprogramming (Fig. 1G and H). A method was then established to use DNA methylation (DNAm) to detect lineage trans-differentiation in human clinical tumor samples. Inspired by the observation that cancer cells switch to transcriptionally distinct fates resembling cells from alternative organs, non-negative least squares (NNLS) deconvolution was performed on the DNAm profiles of samples against known normal tissue DNAm profiles [101], yielding a vector of “tissue similarity scores” for each sample (Fig. 4A).

This method was tested on 35 primary LUAD resection specimens from an in-house cohort (Challenge-Lung). Based on their DNAm similarity to a panel of normal tissue methylation references, the tumors were segregated into 4 groups: low-grade (LG), mid-grade (MG), high-grade (HG), and trans-differentiated (Trans) (Fig. S17A). Notably, this classification aligned with pathological grade (Fig. S17A and B): The LG group mostly consisted of low-grade G1/2 lepidic and acinar cancers [102], the MG and HG groups contained high-grade G3 cancers, and the Trans group included histologically non-adenocarcinoma cancers, such as small cell lung carcinoma and lung adenosquamous carcinoma, likely transformed from adenocarcinoma precursors. This segregation was largely attributed to the loss of lung-specific DNAm profiles and the acquisition of non-lung epithelial DNAm profiles (Fig. S17C). Importantly, since the DNAm analysis was performed on primary tumor samples, the presence of non-lung DNAm profiles is unlikely to result from contamination by non-lung normal cells, but rather from the transformation of cancer cells into alternative lineages.

From the cancer methylation deconvolution profiles, a DNAm deviation index (DNAmDev index) was derived, defined as the difference between the DNAm weight of non-origin epithelial tissue and the original tissue (Fig. 4A). As expected, the DNAmDev index increased from LG to MG and HG, peaking in the Trans group (Fig. 4B). This finding was validated by analyzing the external TCGA (The Cancer Genome Atlas) LUAD cohort [39]. In the TCGA LUAD cohort, the DNAmDev index correlated with histopathological grade, with more aggressive histopathological types exhibiting higher DNAmDev index values (Fig. S17D). Correlation of the DNAmDev index with RNA expression profiles in the same samples revealed that the DNAmDev index was negatively correlated with lung-specific TFs such as *NKX2-1* and *CEBPA*, and positively correlated with non-lung, alternative-lineage TFs like *SOX9*, *HNF4A*, *CDX1*, and *PDX1* (Fig. 4C). Thus, the DNAmDev index provides a means to detect lineage trans-differentiation in human cancer.

To validate whether trans-differentiation is associated with metastasis in human cancers, the DNAm profile of primary LUAD and brain metastases [103] was decomposed against a normal reference panel, revealing a significant increase in non-lung DNAm signals in the metastases (Fig. 4D). This result was further validated by profiling DNAm in an in-house tumor sample cohort (Challenge-PANCAN, *N* = 44 samples from 24 patients, including 12 with paired primary-metastatic tumor samples). An increase in non-origin alternative tissue DNAm signals was observed in all tested primary-metastasis pairs across 5 tumor types, including LUAD, STAD, PRAD, meningioma (MEN), and pancreatic neuroendocrine tumor (PNET) (Fig. 4E and F). This suggests that trans-differentiated cells are more prevalent in metastases than in primary sites, and this phenomenon is not exclusive to LUAD.

In a patient with prostate cancer, primary adenocarcinoma, neuroendocrine-transformed foci in the prostate, and liver metastatic lesions were sequenced (Fig. 4F). The liver metastatic clone was derived from the neuroendocrine-transformed clone in the prostate, as both lesions shared a similar RB1 mutation. Nonprostate DNAm signals increased from the primary to the premetastatic site, and further increased in the liver metastases. Similarly, using cell replicational age estimation from scATAC data, it was found that late-derived trans-differentiated cancer cell clones dominated the metastases (Fig. S18) in a human CRC PDX model [104]. These findings suggest that epigenomically trans-differentiated cancer clones are responsible for seeding metastasis in various cancer types.

Tumor DNAm profiles in the TCGA cohort (*N* = 9,837 samples) [105] were systematically analyzed using this method (Fig. S19A). The DNAmDev index varied across cancer types (Fig. S19B), with tumor types exhibiting higher DNAmDev indices showing significantly worse overall survival (OS) compared to those with lower DNAmDev values (Fig. S19C).

A previous study used machine learning to develop a DNAm-based stemness scoring algorithm (mDNAsi) for tumors, which measures the similarity between the observed DNAm profile and that of embryonic stem cells [68]. In the TCGA cohort, the DNAmDev index showed inconsistent correlation with mDNAsi (Fig. S20), indicating that the 2 indices represent distinct biological processes: The DNAmDev index likely reflects trans-differentiation, while mDNAsi is more likely associated with a fetal-like state. In the TCGA cohort, regional remission and distal metastases were strongly associated with an increased DNAmDev index (Fig. S21A), but only weakly with mDNAsi (Fig. S21B). In multivariate Cox regression analysis (*N* = 8,890 samples with available survival data), including DNAmDev, mDNAsi, and clinical stage, the DNAmDev index was the only factor contributing to the progression-free interval (PFI) (Fig. S21C), and it independently predicted worsened OS regardless of tumor clinical stage (Fig. S21D). Among various cancer types, including bladder cancer (BLCA), LUAD, kidney clear cell carcinoma (ccRCC), PRAD, and low-grade glioma (LGG), patients with high DNAmDev tumors exhibited significantly worse progression-free survival (Fig. 4G). In summary, these results indicate that lineage trans-differentiation primes metastasis and progression in human cancer.

### Genetic gain of oncogenic MAPK signaling associates with lineage trans-differentiation

**Fig. 5.**
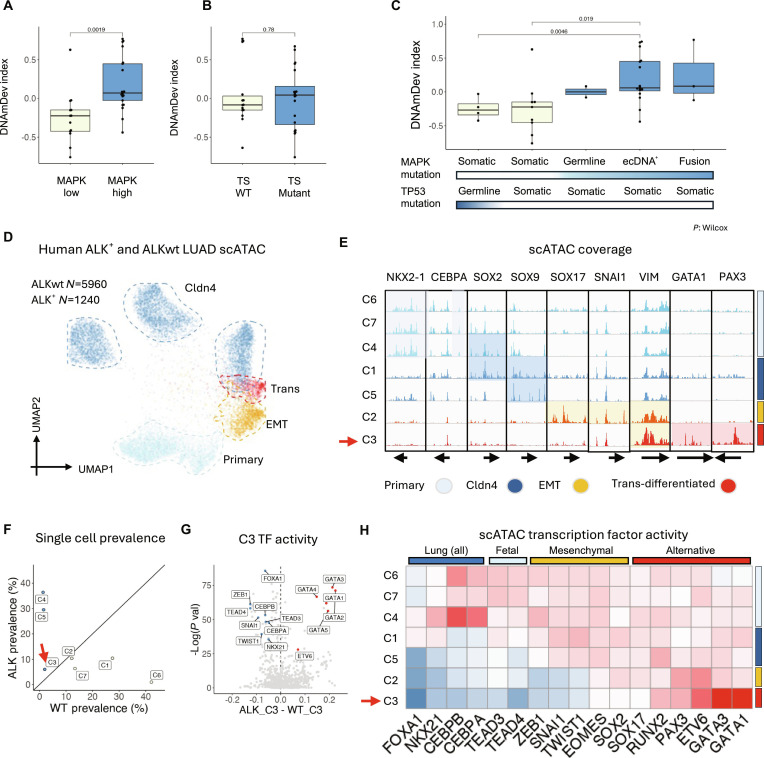
Genetic gain of MAPK signaling associates with lineage trans-differentiation. (A) DNAmDev index of cancers with high and low MAPK activity. MAPK high: carrying germline pathogenic MAPK mutations, extrachromosomal MAPK amplification, or MAPK fusions. (B) DNAmDev index of tumor suppressor-mutated and wild-type cancers. (C) DNAmDev index of cancer samples segregated by TP53 mutation (somatic or germline) and MAPK mutation [somatic, germline, extrachromosomal DNA (ecDNA^+^), or fusion via structural variation]. (D) UMAP of human ALK^wt^ and ALK^mut^ scATAC cells. (E) Chromatin accessibility around marker genes for each scATAC cell cluster. Arrow indicates the trans-differentiated cluster. (F) Relative prevalence of each scATAC cell cluster in ALK^wt^ and ALK^mut^ samples. Arrow indicates the trans-differentiated cluster. (G) Differential transcription factor activity between ALK^wt^ and ALK^mut^ scATAC cells of the trans-differentiated cluster. (H) Transcription factor activity profile in each scATAC cell cluster. *P* value: Wilcoxon test.

The association between genetic mutations and the DNAmDev index was systematically evaluated in the Challenge-Lung cohort samples (Fig. S17A and Table S2). Since the sample size is limited, we reasoned that single gene analysis is unlikely to yield significant association. Therefore, we also gathered oncogenic MAPK alterations [germline pathogenic epidermal growth factor receptor (EGFR) mutations, MAPK oncogene amplification on extrachromosomal DNA (ecDNA) [106], and structural variants leading to high-expression MAPK fusions (kinase fusions) (Fig. S1)] or tumor suppressor mutations (including p53, CDKN2A/2B/1A, RB1, KEAP1, and APC) as a group for analysis. Aside from the gain-of-function in MAPK signaling, no other genetic alterations were found to be significantly linked with the DNAmDev index. Particularly, a strong gain in MAPK signaling (MAPK-high) was more prevalent in the HG and Trans groups. MAPK-high tumors exhibited a significantly increased DNAmDev index compared to MAPK-low tumors (Fig. 5A). In contrast, the loss of tumor suppressors was not associated with changes in the DNAmDev index (Fig. 5B).

In the Challenge-Lung cohort, 4 patients carried germline TP53 loss-of-function mutations, and 2 patients had pathogenic (oncogenic/activating) germline EGFR mutations. Loss of tumor suppressors prior to somatic MAPK mutations in germline TP53 variant carriers did not increase the DNAmDev index (Fig. 5C). In contrast, germline EGFR mutations led to an increase in the DNAmDev index (Fig. 5C). Additionally, in 3 patients with STAD exhibiting brain metastases from the Challenge-PANCAN cohort, the copy numbers of MAPK oncogene amplification on ecDNA not only increased along the evolutionary trajectory but also correlated with lineage trans-differentiation, as indicated by the DNAmDev index (Fig. S22).

To further validate these findings, scATAC-seq data from EML4-ALK fusion-positive and fusion-negative human LUAD were analyzed (Fig. 5D and Fig. S1) [107]. Trans-differentiated cancer cells, which express alternative-lineage TFs (Fig. 5, E and H), were found to be more enriched in EML4-ALK^+^ cancers (Fig. 5F). Moreover, within the trans-differentiated cancer subcluster, the activity of alternative-lineage TFs was significantly higher in EML4-ALK^+^ cells, while lung-specific TF activity was notably reduced (Fig. 5G). Similarly, in the scRNA dataset from the same tumors, EML4-ALK^+^ cells exhibited decreased expression of fetal-like and EMT-related genes, coupled with an increase in expression of genes from alternative lineages (Fig. S23). These results suggest that in vivo trans-differentiation is linked to the genetic activation of MAPK signaling.

MAPK signaling downstream of RTK promotes de novo DNAm [108–111]. The DNAm surrounding TF-binding sites (TFBS) across the genome was computed for tumors in the Challenge-Lung cohort. MAPK-high tumors exhibited higher DNAm levels around most (88%) TFBS compared to MAPK-low tumors (Fig. S24A and B), indicating that the gain of MAPK signaling induces global de novo DNAm in cancer, as has been previously observed in other tissues. Significantly hypermethylated TFBS in MAPK-high tumors affected NKX2-family, TP53, and EMT-related TFs (Fig. S24C), suggesting that MAPK signaling activation may down-regulate lung-like and EMT gene programs by inhibiting their respective driver TF functions. In contrast, the loss of tumor suppressors did not appear to affect DNAm at TFBS related to trans-differentiation (Fig. S24D).

Our computational analysis identified the alternative-lineage TFs ETV6, GATA1, GATA3, SOX2, and SOX17 as up-regulated in the trans-differentiated cells in MAPK-activated lung cancer (Fig. 5H). To functionally validate whether these alternative-lineage TFs drive metastasis-associated phenotype in these cells, we performed small interfering RNA (siRNA)-mediated knockdown of each TF in the human lung cancer cell lines PC9 and A549. Transwell migration and invasion assays revealed that silencing these trans-differentiation TFs significantly impaired the migratory and invasive capacities of both cell lines compared with control siRNA-treated cells (Fig. S25).

These results provide experimental evidence that lineage trans-differentiation is functionally required for the invasive phenotypes associated with metastasis. This complements our computational findings and strongly supports the conclusion that lineage trans-differentiation is a driver, not just a passenger, of metastatic progression.

### MAPK inactivation reverses human cancer lineage trans-differentiation

**Fig. 6.**
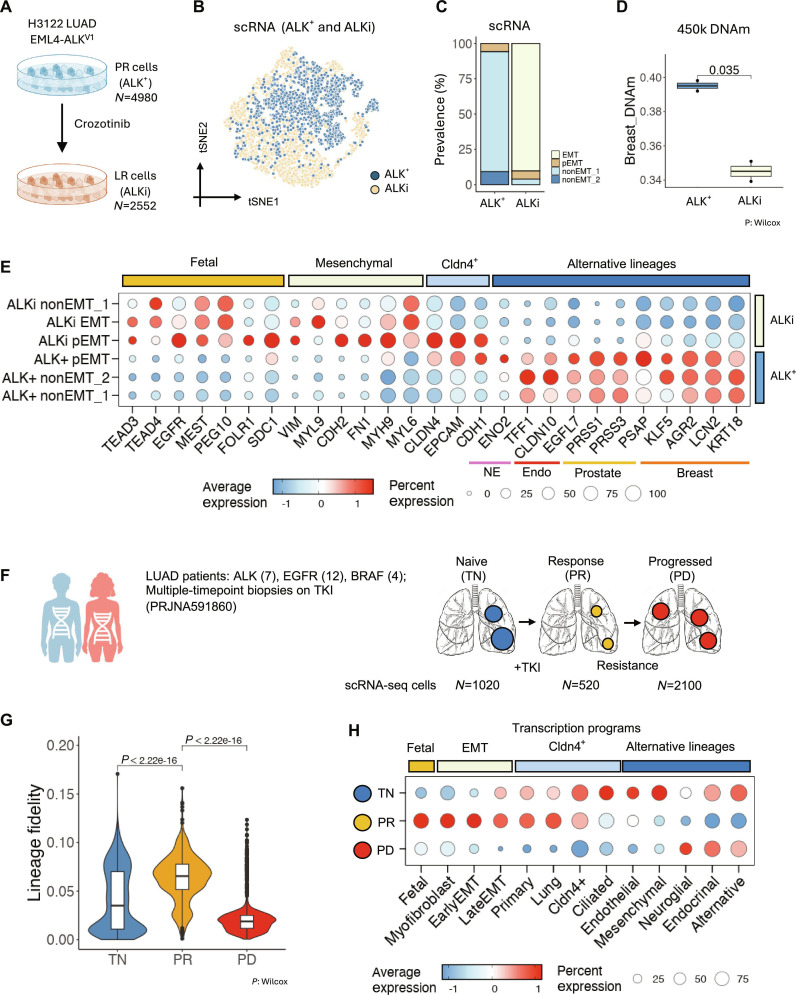
MAPK inactivation reverses human cancer lineage trans-differentiation. (A) Schematic of the experiment to determine gene expression profile changes after ALK inhibitor application in the EML4-ALK^+^ human LUAD cell line H3122. (B) UMAP of ALKi H3122 cells and ALK^+^ treatment-naive H3122 cells. (C) Prevalence of each cell type in ALKi and ALK^+^ cells. (D) Similarity to breast DNAm profiles of ALKi and ALK^+^ cells. (E) Key gene expression profile in ALKi and ALK^+^ cells. (F) Schematic of the experiment to determine gene expression profile changes after TKI application in human cancers in vivo. (G) Lineage fidelity score of treatment-naive (TN), response stage (PR), and progression stage (PD) biopsy single cells. (H) Gene program expression profile in TN, PR, and PD biopsy single cells. *P* value: Wilcoxon test.

The functional requirement of MAPK signaling for post-EMT lineage trans-differentiation was tested by analyzing gene expression and DNAm changes following ALK pharmacological inhibition in an EML4-ALK^+^ human cancer cell line in vitro (Fig. 6A and B). ALK inhibition in these cells led to a switch from an epithelial-like phenotype to an EMT-like phenotype (Fig. 6C), a reduction in alternative-lineage DNAm profiles (Fig. 6D), decreased expression of alternative-lineage genes, and the re-induction of fetal-like and EMT-related genes (Fig. 6E).

To determine whether this phenomenon applies to other MAPK drivers, 7 bulk RNA-seq datasets of EGFR- and RET-driven lung cancer cells treated with tyrosine kinase inhibitors (TKIs) were systematically evaluated (Fig. S26A). Gene set enrichment analysis (GSEA) revealed that acute TKI application induces the activation of myogenic and EMT gene sets, with this activation maintained in TKI-resistant cell lines selected by chronic TKI exposure (Fig. S26B). In fetal lung-tip organoids, treatment with extracellular signal-regulated kinase (ERK) or MAPK kinase (MEK) inhibitors, but not AKT inhibitors, similarly induced myogenic and EMT gene programs (Fig. S26B), consistent with the understanding that oncogenic RTK signaling primarily activates ERK/MEK rather than AKT [36]. Collectively, these findings suggest that oncogenic RTK signaling through ERK or MEK is necessary for the lineage trans-differentiation of EMT cancer cells.

This result was further validated using a human clinical dataset of LUAD scRNA data from pre-TKI (naive, TN) and post-TKI (response, PR; and TKI-resistant progression, PD) biopsies [112] (Fig. 6F). In these cells, TKI treatment was associated with increased lineage fidelity, while disease progression was linked to a reduction in lineage fidelity (Fig. 6G). TKI response resulted in the suppression of alternative-lineage-specific transcription programs and the activation of fetal and EMT-related programs (Fig. 6H). These events were reversed in the TKI-resistant progression samples. Together, these data demonstrate that MAPK signaling drives post-EMT lineage trans-differentiation.

## Discussion

**Fig. 7.**
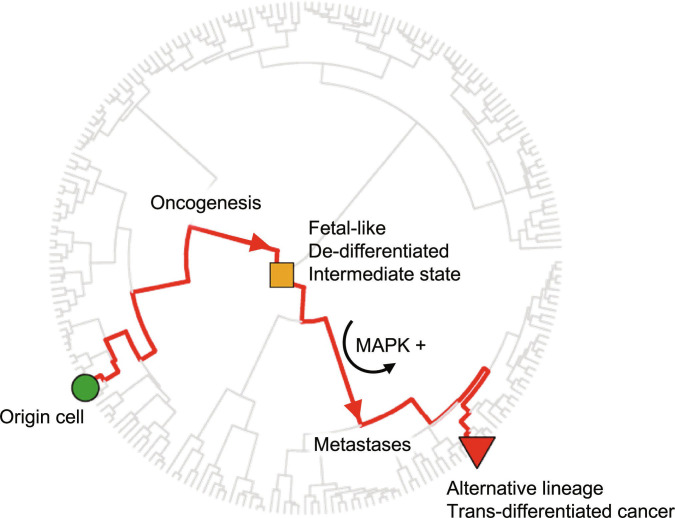
Summary of findings: Evolution of cancer metastases through trans-differentiation. The minimal transition trajectory (red) between the origin lung AT2 cell and an observed LUAD single cell, which has adopted an alternative-lineage normal cell, is shown on a reference normal cell phylogenetic tree (plotted in a circular structure, with the most ancestral/developmentally immature cell at the center and terminally differentiated cells at the outer edge). The metastatic cancer cell first evolves from the origin normal cell through a series of de-differentiation steps toward a fetal-like state and then trans-differentiates to adopt an alternative-lineage fate, enabling the formation of metastases. MAPK-driven cancers rely on MAPK signaling for trans-differentiation.

Through integrated scRNA and scATAC analyses of the mouse LUAD model, we characterized the evolutionary trajectory from primary cancer to metastatic-ready clone, and found that cancer cells first undergo de-differentiation during early oncogenesis before EMT, by down-regulating cell-of-origin-specific transcription factors and their downstream targets. These premetastatic cells then re-differentiate to adopt alternative-lineage transcriptional programs, by mis-expressing and activating alternative-lineage transcription factors. These results are further supported by scRNA, scATAC, and DNAm analyses across multiple human cancer types, including primary samples, organoid systems, and xenograft models. Thus, fetal-like primitive progenitor state primarily functions as a gateway to trans-differentiated cancer. De-differentiated EMT cells, characterized by a fetal signature, act as precursors for metastases. These cells subsequently re-differentiate, adopting alternative-lineage gene expression profiles, thereby forming trans-differentiated metastatic clones (Fig. 7).

Lineage trans-differentiation has been observed in various cancer types, in both mouse models and human clinical samples. Clinically, lineage trans-differentiation is strongly associated with histopathological grade, metastatic potential, and poor clinical prognosis. Although the presence of a fetal-like state predicts future trans-differentiation, it represents a transient intermediate stage during metastatic cancer evolution and may be absent in advanced-stage endpoint samples, such as tumor biopsies. Therefore, using the fetal-like state alone for clinical prediction may lead to false negatives. In contrast, the presence of a trans-differentiated state in endpoint tumors indicates that a fetal-like state likely existed during the tumor’s evolutionary history. This temporal relationship may explain why lineage trans-differentiation outperforms the fetal-like state in predicting clinical outcomes.

While fetal-like states are shared among tumors, best characterized by up-regulation of the EMT-associated and myogenic transcriptional program and global activation of chromatin, the trans-differentiated states are more diverse. On the single-cell level, each trans-differentiated cancer cell adopts a distinct state (Fig. S13B). Occasionally, we observe intermediate-state cells showing transcriptional similarities to more than one cell type, and the developmental distance between the target cell types increases as tumor stage progresses (Fig. S13C and D). These observations suggest that the “off-target” lineage signatures may reflect a generalized state of high cellular plasticity rather than a shared common signature across metastatic cancers.

While the activation of the MAPK pathway has been extensively studied in early-stage oncogenesis [54,113–115], its role in promoting metastasis remains less clear. Our results indicate that lineage trans-differentiation is associated with the genetic gain of MAPK signaling and can be reversed by MAPK inhibition. Specifically, in human LUAD, alternative-lineage transcription factors are more active in EML4-ALK^+^ cells than in wild-type cells (Fig. 5D to H), suggesting that elevated oncogenic MAPK signaling is linked to acquisition of the trans-differentiated program. Consistently, pharmacological inhibition of ALK in an EML4-ALK^+^ human cancer cell line suppresses alternative-lineage genes and restores fetal/EMT-associated programs at both the transcriptomic and DNA methylome levels (Fig. 6A to E). Extending beyond ALK-driven tumors, we re-analyzed additional EGFR- and RET-driven cancer datasets and observed that MAPK-targeting TKI treatment reproducibly shifts tumor cells toward a less trans-differentiated, more lineage-faithful state (Fig. S26). Crucially, this pattern is also evident in paired patient biopsies: TKI exposure in vivo is associated with increased lineage fidelity, whereas subsequent resistance and progression coincide with its loss (Fig. 6F to H). Although direct causal evidence in human tumors is still lacking, these convergent observations strongly suggest that oncogenic MAPK signaling is necessary to maintain, and may drive or stabilize, lineage trans-differentiation. Interventions targeting this molecular pathway could therefore potentially prevent cancer metastasis. In clinical trials, postoperative adjuvant TKI treatment has proven beneficial in preventing metastasis in driver MAPK mutation-positive LUAD, BRCA, and melanoma individuals, as demonstrated in the ADAURA, ALINA, COMBI-AD, NEfERT-T, and NALA trials [116–121]. Our findings not only help explain these results but also suggest future directions for developing and improving clinical interventions to combat metastasis.

The adoption of an alternative-lineage fate through epigenomic reprogramming may provide a selective advantage during metastatic seeding. Consistent with the population bottleneck we observed at the early metastatic stage (EMT/Met1) (Fig. 1K), trans-differentiation could expand ITH and generate phenotypic variants that better survive immune attack and treatment, while alternative-lineage transcriptional profiles may enhance adaptation to distal metastatic niches. In this context, trans-differentiated cells might become locked in a partially reprogrammed, intermediate progenitor-like epigenomic state, enabling both increased motility and aggressive proliferation, which together could underlie their high metastatic potential. We additionally considered whether such “off-target” lineage programs dictate organ tropism. Although DNAm deconvolution revealed distinct non-self lineage methylation patterns across different cancer types (Fig. S19), we did not establish a clear causal link between these patterns and metastatic site preference. For instance, glioblastoma (GBM) and LGG tumors sometimes exhibit pancreatic-, breast-, or prostate-like methylation patterns, which do not correspond to their typical sites of metastasis. However, this does not exclude the possibility that metastatic niche preference is determined by the trans-differentiation state at the transcriptional level. In support of this hypothesis, while this paper is under review, another group reported that pancreatic adenocarcinoma cells adopting lung- or liver-parenchymal-like transcriptional features preferentially form metastases in the corresponding organs [122]. Together with our prior finding that trans-differentiation can facilitate immune evasion by down-regulating immunogenic molecules and up-regulating immunosuppressive programs [123], these observations support a model in which trans-differentiation promotes metastatic fitness through niche adaptation, immune escape, and a hybrid motility-proliferation phenotype. Future in vivo perturbation and lineage-tracing experiments will be required to test these hypotheses directly.

The current study has some limitations. Our data are primarily based on re-analysis of published datasets and lack direct manipulation experiments demonstrating that inducing trans-differentiation converts nonmetastatic cancer into metastatic cancer, which should be addressed in future studies. The re-analysis of a large number of publicly available datasets from different laboratories and technologies may introduce inconsistencies and potential false negatives. While our DNAm analysis provides an important layer of epigenomic evidence linking MAPK signaling to cell fate regulation in metastasis, histone modifications and higher-order chromatin structure were not analyzed, which are beyond the scope of the current work. Moreover, although our data demonstrate that MAPK signaling is necessary to maintain the trans-differentiated state, future genetic perturbation and lineage-tracing experiments, such as activating and inactivating MAPK to change metastatic potential of cancer cells, are needed to confirm whether MAPK signaling is sufficient to drive this process. As our study primarily relies on bioinformatic analysis, the results have not been validated through reverse genetics in animal models. Finally, this study did not investigate whether trans-differentiation enables metastatic cancer cells to adapt to a non-native metastatic microenvironment, evade immune surveillance, or enhance their mobility in vivo. These questions should be addressed in future experimental studies.

## Materials and Methods

### Human biospecimens

Clinical information and samples (including blood and surgical tissue samples) were collected from the multi-center, prospective, observational cohort for cancer, known as the Cancer HALLmark Epigenetics aNd Genetics (CHALLENGE). The study included 35 samples (*n* = 35, from 35 donors) from the lung cancer subcohort (Challenge-Lung) and 44 samples (from 24 patients) from the pan-cancer cohort (Challenge-PANCAN). These samples were collected from Wuhan and Beijing, China, and were processed and preserved by the Hubei Biobank, an official member of the International Society for Biological and Environmental Repositories. The study received approval from both the Institutional Ethics Committee of Zhongnan Hospital of Wuhan University (approval numbers: 2017038-1 and 2024061K) and the China Human Genetic Resources Management Office, Ministry of Science and Technology of China (approval number: 2022BC0128). Written informed consent was obtained from all participants involved in the study.

### Public sequencing datasets

This study incorporated a large number of publicly available sequencing datasets from previous research. A comprehensive list of these datasets can be found in Table S3.

### Histopathology of clinical cancer samples

For hematoxylin and eosin (H&E) staining, formalin-fixed, paraffin-embedded (FFPE) tissue sections were deparaffinized in xylene and rehydrated through a graded ethanol series. Nuclei were stained with hematoxylin (5 min), followed by differentiation in 1% acid ethanol (30 s) and bluing in running tap water (5 min). Cytoplasmic counterstaining was carried out with eosin (2 min). Sections were then dehydrated through graded ethanol, cleared in xylene, and mounted with neutral resin. All procedures were performed at room temperature, and stained slides were evaluated using bright-field microscopy.

### Cell culture

Human lung cancer cell lines PC9 and A549 were kindly provided by Cell Bank, Chinese Academy of Sciences (Shanghai, China). PC9 and A549 cells were cultured in RPMI 1640 supplemented with 10% fetal bovine serum (FBS) and 1% penicillin–streptomycin (10,000 U/ml). Each cell line was authenticated and tested negative for mycoplasma contamination.

### Cell transfection, RNA extraction, and qRT-PCR

siRNAs were purchased from GenePharma (Shanghai, China). The target sequences of the siRNAs used in this study are listed in Table S4. Cells were transfected with siRNAs using Lipofectamine 3000 (Invitrogen, L3000150) according to the manufacturer’s instruction. Total RNA was isolated with the HiPure Total RNA Mini Kit (Magen, R4111-03). cDNA was synthesized using the ReverTra Ace qPCR RT Kit (TOYOBO, FSQ-101). Quantitative reverse transcription polymerase chain reaction (qRT-PCR) was performed on StepOnePlus real-time PCR system (Thermo Fisher, USA) with SYBR Green SuperMix (Bio-Rad, 1725125). Primer sequences are provided in Table S4.

### Cell migration and invasion assays

Cell migration and invasion were evaluated using 24-well Transwell inserts (Falcon, 353097). For the migration assay, 5 × 10^4^ PC9 cells or 6 × 10^4^ A549 cells were resuspended in 200 μl of serum-free RPMI 1640 media and seeded into the upper chambers. For the invasion assay, 1 × 10^5^ PC9 cells or 2.1 × 10^5^ A549 cells were resuspended in 200 μl of serum-free RPMI 1640 media and added to the upper chambers that had been precoated with Matrigel (Yeasen, 40183ES08). In all experiments, 600 μl of RPMI 1640 supplemented with 20% FBS was added to the lower chambers as a chemoattractant. The Transwell chambers were incubated for 24 h to allow cells to migrate or invade through the porous membrane. After incubation, nonmigrated or non-invaded cells remaining on the upper surface of the membranes were carefully removed with a cotton swab. Cells that had traversed the membranes and adhered to the lower surface were fixed in 4% paraformaldehyde and stained with crystal violet.

### scRNA data preprocessing, stemness inference, and cross-dataset integration

The analyzed scRNA data were presented as gene-x-cell count matrices. Datasets were processed individually using Seurat (4.1.3) for reading, normalization, variable gene selection, PCA, neighbor finding via K-nearest neighbors (KNN), clustering, and uniform manifold approximation and projection (UMAP). Default parameters were used for all preprocessing. The top 30 PCA vectors were applied to all datasets. Differentially expressed gene (DEG) analysis based on single-cell clusters was performed using the presto::wilcoxauc (1.0.0) package. For each cluster, the top 20 to 30 DEGs ranked by area under the curve (AUC) were used for manual annotation of cell types with assistance from the DeepSeek API (https://platform.deepseek.com/). Epithelial cells were selected and cleaned up from non-epithelial stromal and hematopoietic lineage cells by cluster annotation. Since estimation of stemness can be influenced by batch effects, CytoTRACE (0.3.3) analysis was performed individually for epithelial cells from each dataset using default parameters.

For cross-dataset integration, epithelial cells from each dataset were combined using Harmony with default parameters. For both mouse normal and cancerous lung cells, standards set by LungMAP [49] and Marjanovic et al. [41] were adhered to. Rather than breaking down the analysis to a very fine granularity for cancerous cells, they were broadly classified into primary cancer, Cldn4^+^, EMT, and metastasis-associated (Met) cells. The maximum number of datasets contained cells from all of these clusters.

### Inferring the likely cell type of last common ancestor and estimating progenitor population size in scRNA lineage-tracing data

The occurrence timing of single-cell clusters (AT2, BASC, Primary cancer, Cldn4^+^, EMT, Met) was determined by pseudotime analysis using Slingshot (2.4.0) within the integrated dataset. The phylogenetic tree derived from the scRNA lineage-tracing data [43] was provided by the authors on Zenodo (see Data Availability statement). Single-cell type annotations were used as leaf labels. The tree was recursively backtracked from each leaf, and at each node, the node was annotated by the most ancestral (early-emerged) label among its leaves. This process was completed across the tree to determine the ancestor cell type for each observed single cell (leaf).

To estimate the total progenitor population from a given cell type within these samples, each sample was processed individually using TarCA (TarCA.beta, 0.7.0) following the authors’ guidelines. The Np value estimated by TarCA was used to represent the progenitor size (Table S1).

### Transcription module extraction from scRNA (Hotspot)

The scRNA-seq count matrix was exported from the integrated Seurat object and saved as h5ad using MuDataSeurat (0.0.0.9000) in R (4.1.3), then read in Python (3.9.7) with Scanpy (1.10.1). The data were normalized and log1p-transformed in Scanpy using default parameters. Genes with mean expression within ±5 SDs and dispersions ≥0.5 were selected. The top 3,000 variable genes from this selected set were used and combined with known markers (e.g., *Nkx2-1*, *Cebpa*, *Cebpe*, *Cebpb*, *Foxa1*, *Foxa2*, *Sox17*, *Sftpb*, *Sftpc*, *Sftpa1*, *Scgb1a1*, *Scgb3a2*, *Scgb3a1*, *Clu*, *Hopx*, *Pdpn*, *Ager*, *Cyp2f2*, *Sox2*, *Cldn4*, *Apoe*, *Cd24a*, *Gdf15*, *Tm4sf1*, *Slc4a11*, *Onecut2*, *Vim*, *Sox9*, *Lgals1*, *Zeb1*, *Zeb2*, *Snai1*, *Snai2*, *Twist1*, *Twist2*, *Id3*, *Gpx3*, *Bmp7*, *Runx1*, *Runx2*, *Gata3*, *Gata1*, *Gata5*, *Hnf4a*, *Pdx1*, *Cdx2*, *Osr1*, *Prrx1*, *Meis1*, *Meis2*, *Myc,* and their human orthologs). Hotspot (1.1.1) was used to run the analysis with the parameters “n_neighbors = 30” for KNN graph creation, “FDR < 0.05” for marker gene selection, and “min_gene_threshold = 30, core_only = False, fdr_threshold = 0.05” for module creation. Module expression scores for each single cell were generated automatically by Hotspot. Manual annotation of each module was done with assistance from the DeepSeek API.

### Inferring scRNA cell lineage fidelity using correlation

Both the raw scRNA count matrix (“observation”) and the respective reference normal cell matrix (“reference”) (mouse: Mouse Cell Atlas; human: Human Protein Atlas single-cell RNA expression) were depth-normalized to a total of 1e+5 and log1p-transformed. Pearson correlation was performed using WGCNA::cor (1.71) between the observation and reference matrices. For each single cell, the top 10 correlation coefficients were extracted, and lineage fidelity was estimated as follows: stdev(cor_coef)/mean(cor_coef).

### Single-cell annotation and lineage fidelity inference using Azimuth

The pan-human Azimuth API (0.1.0) with the DISCO database [124] was employed to annotate the human LUAD scRNA dataset, using default parameters. After annotation, the final_level_softmax_prob parameter for each cell was extracted as the confidence level of the prediction.

### Single-cell annotation with probability estimation using maximal likelihood estimation

To calculate lineage fidelity, the observation-x-reference matrix correlation was performed, as described in the Inferring scRNA cell lineage fidelity using correlation section. For each single cell in the observation data, reference cells with the top 10% correlation score were selected as candidate reference hits. The depth-normalized read count of each gene from these candidate reference hits was used to construct the prior probability matrix (mu_matrix). Gene-specific read depth dispersions were estimated from this prior probability matrix, and the size parameter (size_matrix) was calculated as 1/dispersion. The raw read count of each gene for the observation single cell was treated as the observation vector, with the distribution density following a negative binomial distribution. In a vectorized form, the likelihood estimation was performed as follows: logP_density_mat <- dnbinom(counts_matrix, size = size_matrix, mu = mu_matrix, log = T) and log_likelihoods <- colSums(logP_density_mat).

This calculation provided log-likelihoods indicating how similar the observation single cell is to each of the candidate reference cells. These were then converted into probabilities, assuming that the single cell must match one of these candidate references. The top *N* (*N* = 10 in this study) reference cells that accumulated to a probability greater than 95% were selected as the inferred matches for the observed single cell. The top reference cell with the maximal probability was considered the best match to the observed single cell.

### scRNA lineage fidelity inference from maximal likelihood estimation results

To calculate lineage fidelity, a hierarchical clustering of the reference cell gene expression (log1p-transformed) was constructed and transformed into a phylogenetic tree based on gene expression similarity. The total travel distance on this tree between all pairs of matched reference cells for a given observation single cell was calculated as the lineage fidelity index.

### scRNA transformation trajectory from maximal likelihood estimation results

For LUAD, AT2 cells were always assumed to be the ancestor for any cancerous LUAD cells. To plot the minimal transformation trajectory between an observed single cell and its putative ancestor, the expression similarity tree derived from scRNA lineage fidelity inference from maximal likelihood estimation results was used. The most recent common ancestor (MRCA) of the ancestor leaf and the best match cell leaf [as inferred by maximal likelihood estimation (MLE)] was estimated using the ape package (5.7.1). The minimal path joining the ancestor leaf, the MRCA node, and the best match leaf was then drawn. The results were visualized using the ggtree package (3.4.0).

### scATAC data analysis

The scATAC fragment files were downloaded from each public dataset and processed into a unified dataset using ArchR (1.0.1), with the exception of the in vitro lung cancer organoid data, which were processed separately. ArchR analysis was performed using default parameters. After integration, cell types were annotated based on promoter region accessibility of manually curated marker genes. For Cldn4^+^ cell analysis, Cldn4^+^ cells were extracted and fine-level subclustering was performed. Single-cell replicational age estimation and phylogenetic tree construction based on this replicational age were carried out using EpiTrace (0.0.1.3). Cell evolution pseudotime was inferred in ArchR using Monocle. Global accessibility distributions between peak and nonpeak regions were derived from the fragment-in-peak (FRIP) parameter in ArchR. TF activity inference was conducted with ArchR using the cisbp database.

### DNAm sequencing and microarray

DNAm sequencing and data processing for in-house samples were performed on FFPE tumor samples, following the protocol described by Xiao et al. [125]. DNA was extracted from the samples, bisulfite-converted, subjected to poly-adenylation and 3′ adaptor ligation, and then linearly amplified from the 3′ adaptor before ligating to a 5′ adaptor. The PCR-amplified, adaptor-ligated product underwent biotinylated bait capture before sequencing on an Illumina Novaseq platform. The sequencing data were trimmed using fastp (0.19.4) and then mapped to the GRCh37+decoy genome using bwa-meth (0.2.0). Per-CpG DNAm frequency (beta) was calculated with PileOMeth (0.1.13-3). Publicly available DNAm data from the Illumina methylation array were downloaded as IDAT files and processed using minfi (1.42.0) to generate beta matrices. TFBS DNAm analysis was performed by predicting TFBS with ArchR and cisbp, and all reads covering these TFBS were grouped to estimate the average methylation level at each site.

### DNAm-based tissue lineage deconvolution and estimating DNAmDev

NNLS regression was applied to the observation DNAm levels (beta) against the MethAtlas reference panel, specifically for the corresponding differentially methylated loci (DML), using nnls::nnls (1.4), as described in the original paper [126]. The accuracy of the algorithm was first calibrated using publicly available, well-characterized normal control sample data. Including the “normal bladder” reference sample in the reference panel led to erroneous predictions, classifying many nonbladder normal tissue samples as “bladder”. Therefore, bladder samples were removed from the reference panel due to their disproportionate influence on NNLS analysis. The final regression result was not normalized, meaning that the sum of the weights could be less than or equal to 1. The “DNAm deviation” (DNAmDev) index is defined as follows: sum(weight(other_epithelial_tissues)) - weight(cell_of_origin)).

### Bulk RNA sequencing

The RNA-seq data matrix (counts) was either obtained from in-house samples or downloaded and analyzed using DESeq2. GSEA scores for each cluster were derived using the mSigdbR package’s “Hallmark” gene set.

### Somatic mutation sequencing

Somatic mutation sequencing and data processing for in-house samples were conducted on FFPE tumor samples, following the protocols outlined by Xiao et al. [123] and Xue et al. [127]. Only variants clearly annotated as oncogenic mutations and meeting the following criteria were retained: >0.5% variant allele frequency for short nucleotide variants, >3× copy number for amplifications, ≤1× copy number for deletions, or classical kinase fusions with >1% variant allele frequency. ecDNA amplification was called using regions outside of breakage–fusion–bridge cycle (BFB) area or chromothripsis arms, shorter than 10 million base pairs, harboring oncogenes, and with copy numbers >5×.

### Statistics

Multivariate Cox models were employed to analyze OS and PFI. Log-rank tests were used for Kaplan–Meier survival analysis, and 2-sided Wilcoxon tests were used for 2-group comparisons. For box-and-whisker plots of grouped data points, the upper and lower bounds of the boxes represent the 25th and 75th percentiles, respectively, while the median is shown as a horizontal line within the box. The whiskers extend to the minimum and maximum values, defined as the farthest data points within 1.5 times the interquartile range (IQR) from the box bounds. Violin plots represent the empirically estimated density distribution of the data. For enrichment analysis results from Fisher’s exact test, the odds ratio is represented as the median point in the graph, with its 95% confidence interval shown as error bars.

## Ethical Approval

This study was conducted in accordance with the Declaration of Helsinki and received approval from the Institutional Ethics Committee of Zhongnan Hospital of Wuhan University (2017038-1 and 2024061K). Bioinformatics data involving human subjects were sourced from publicly available databases containing anonymized patient information.

## Data Availability

The software packages and their versions used in the study are listed in Table S5. The scLineageFidelity and LineageMLE functions are implemented in R and provided on the GitHub repository (https://github.com/MagpiePKU/scLineageFidelity) together with a comprehensive usage instruction README file. De novo inferred and manually curated gene sets for annotating and scoring single-cell data are provided in Table S6. Publicly available data used in this study can be downloaded from Zenodo (https://zenodo.org/records/5847462, https://zenodo.org/records/7713052), the TCGA official website (https://gdc.cancer.gov), the NCBI website (http://www.ncbi.nlm.nih.gov/), and the MCA website (https://bis.zju.edu.cn/MCA/). In-house generated methylation sequencing data from the Challenge-PANCAN and Challenge-Lung cohorts, used in this study, have been uploaded to the OMIX database at CNGB, China (https://ngdc.cncb.ac.cn/omix/) with accession number OMIX010221 and will be made publicly available upon publication. Due to local legal requirements, the in-house generated human dataset will only be available in processed data format and requires a case-by-case application through the China Human Genetic Resources Management Office. The remaining data are available within the article and in the Supplementary Materials.
