# Supplementary material for: Evolution of Cancer Metastases via Lineage Trans-Differentiation
Source: Research (Wash D C). 2026 Feb 19;9:1144. doi: 10.34133/research.1144 (PMC12917112; doi:10.34133/research.1144)
Supplement: Supplementary 1 — Figs. S1 to S26 Tables S1 to S6 [file research.1144.f1.zip › Supplementary Information.pdf]

Research
A SCIENCE PARTNER JOURNAL
SPJ
SCIENCE PARTNER JOURNALS

Supplementary Materials for

Evolution of cancer metastases via lineage trans-differentiation

Yu Xiao *et al.*

*Lead corresponding author: Yi Zhang, zhangyi@cimrbj.ac.cn

This PDF file includes:

Figures S1 to S26: Pages 2-35
Legends for Tables S1 to S6: Pages 36-37
References (1 to 4): Page 38

Other Supplementary Materials for this manuscript include the following:

Tables S1 to S6

Figs. S1-S26

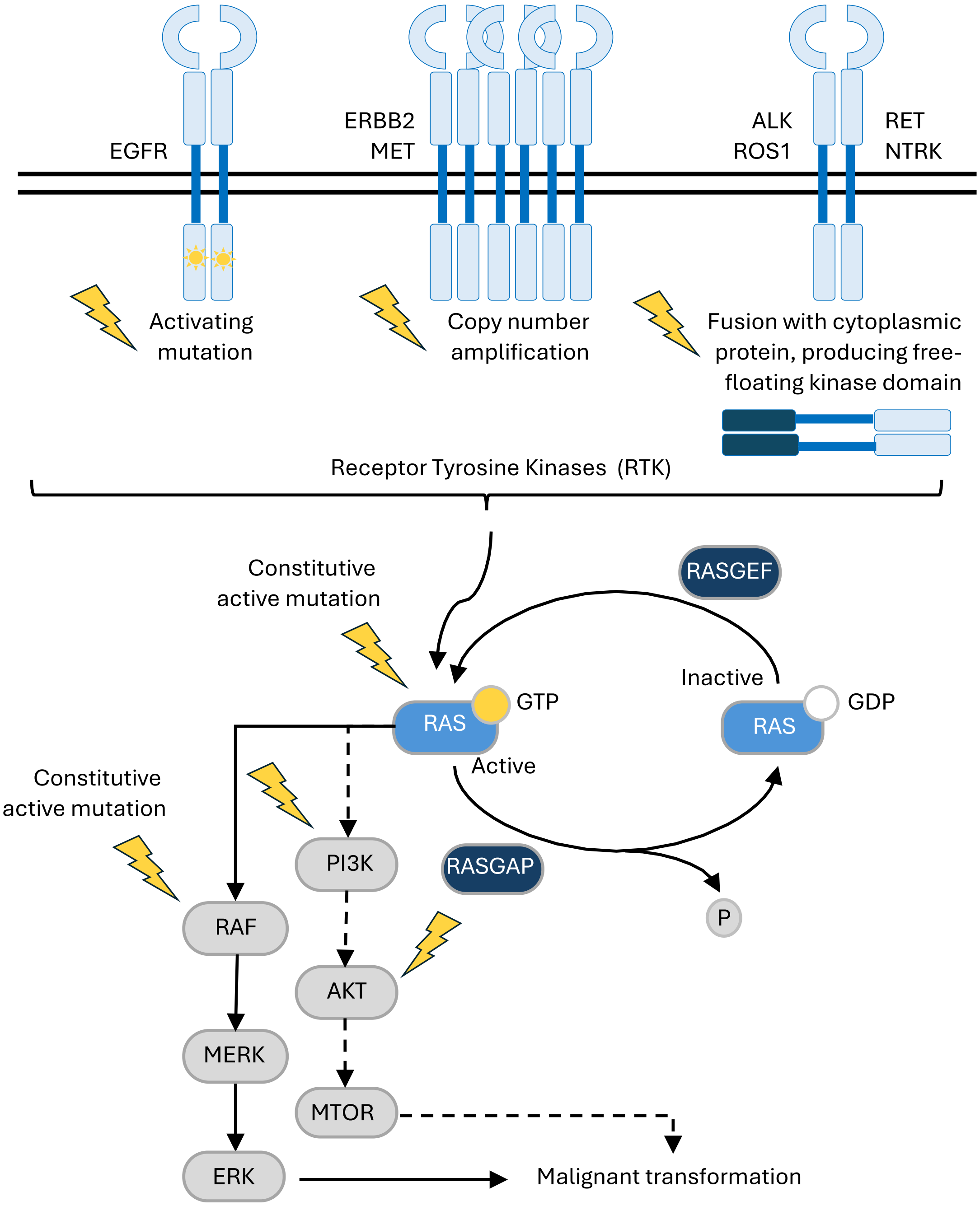

Fig. S1. The RTK-RAS-RAF-MAPK signaling pathway in cancer.
Schematic representation of the RTK-RAS-RAF-MAPK signaling cascade, illustrating key molecular interactions and potential oncogenic mutations that drive cancer cell proliferation, survival, and metastasis. Arrows indicate activation, while blunt-end lines represent inhibition or suppression of downstream signaling events.

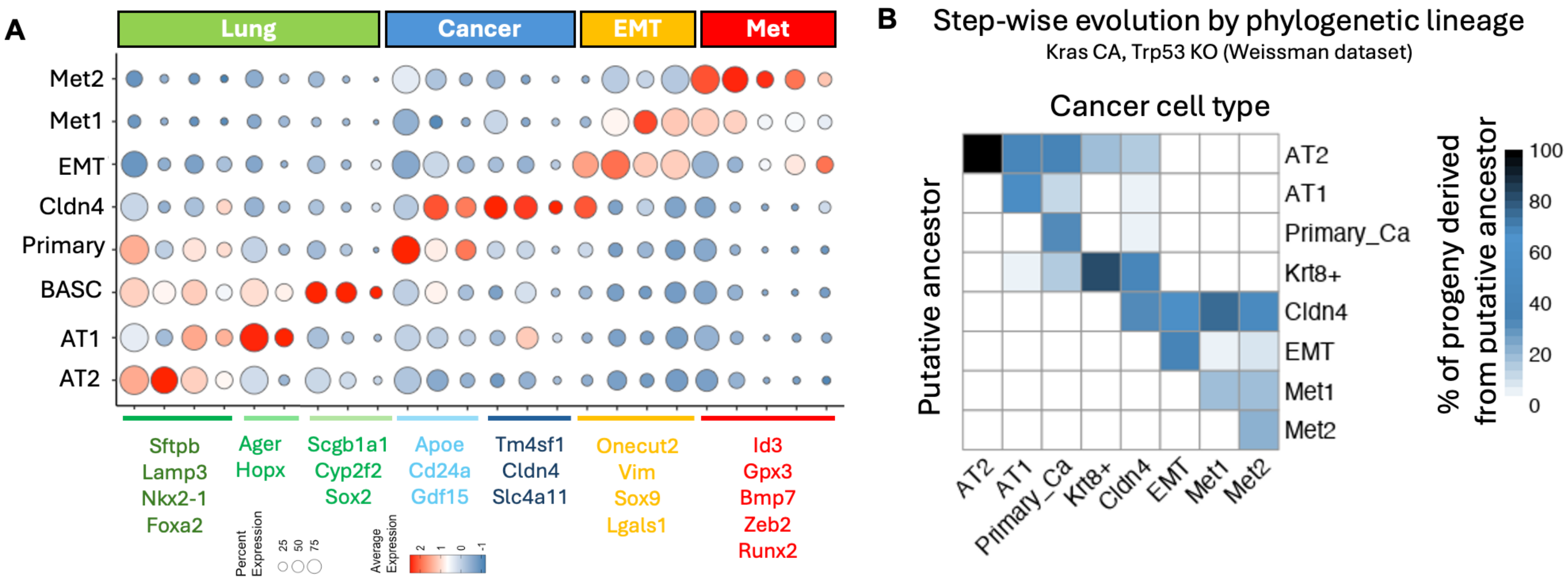

Fig. S2. Tracking tumor evolution in scRNA dataset using genetic lineage tracing.
(A) RNA expression dot-plot of canonical marker genes in each single cell type. **(B)** Percentage of each class of cells (in columns) derived from each class of ancestors (in rows) by summarizing the genetic lineage tracing data (*1*).

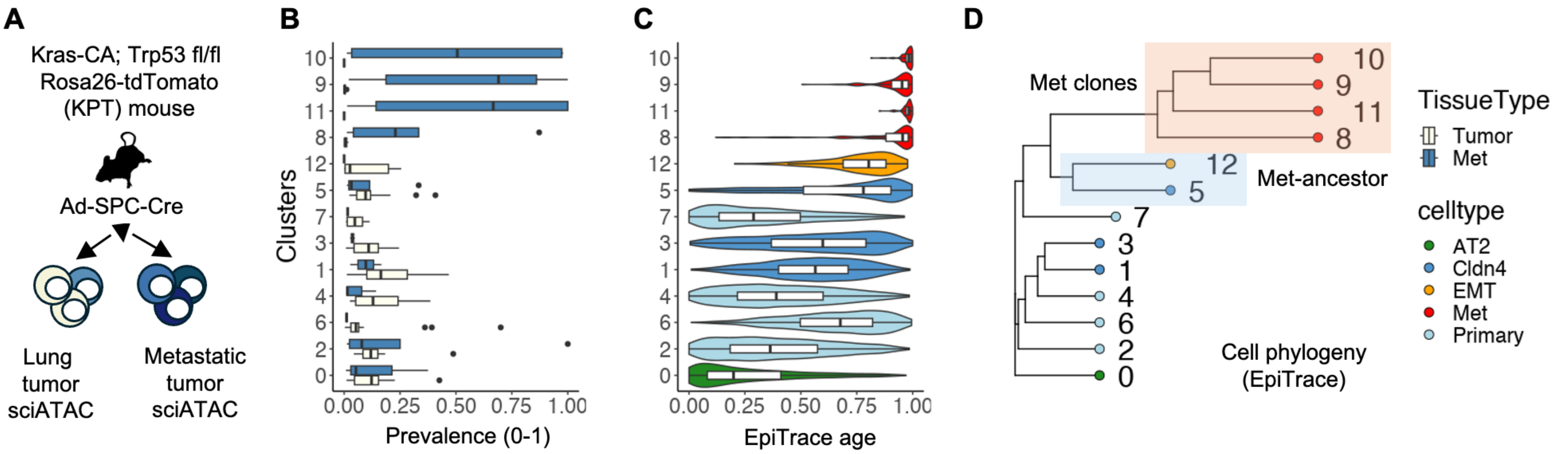

Fig. S3. Tracking tumor evolution in scATAC dataset using single cell age.
(A) Schematic of experimental procedure (adapted from the original publication (*2*)). **(B)** Prevalence of each single cell cluster in primary tumor (white) and metastases (blue) scATAC samples. **(C)** Cell replication age estimated by EpiTrace of each single cell cluster. **(D)** Putative phylogeny of single cell clusters built with clock-like loci by EpiTrace. EMT cells are located in the putative metastatic ancestor branch, and metastases cells form a unique branch from here.

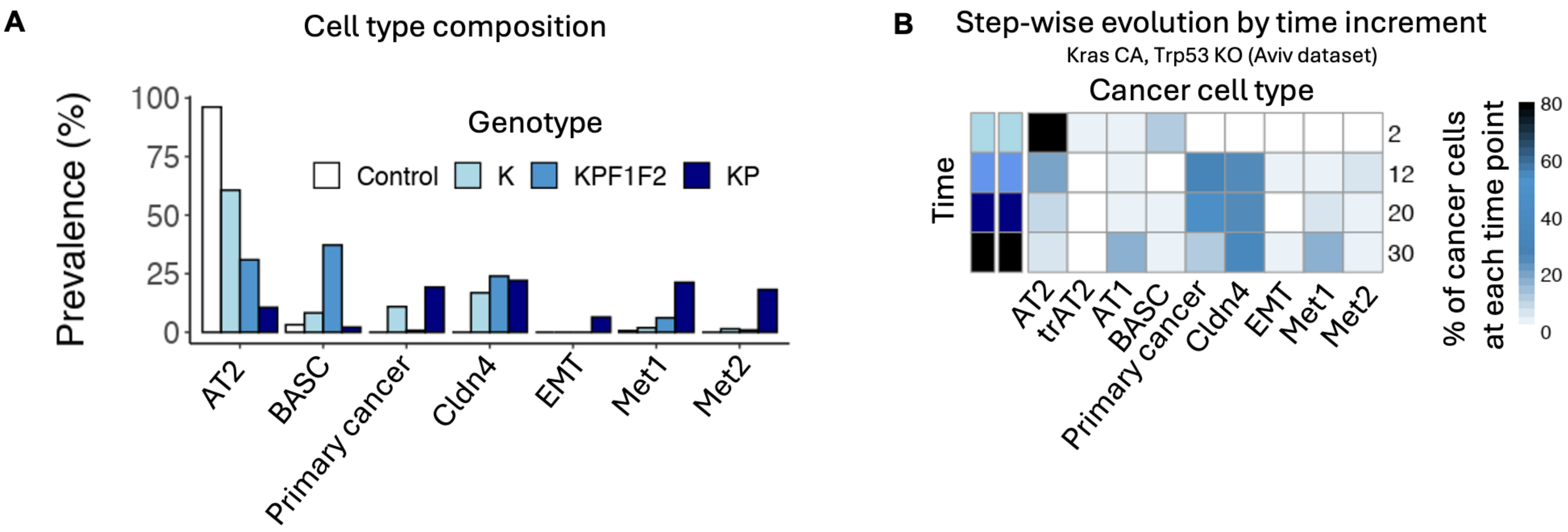

Fig. S4. Lack of requirement of definitive genetic alteration for metastases evolution.
(A) Prevalence of each single cell type from each genotype (Control: wild-type, K: $Kras^{CA}$, KPF1F2: $Kras^{CA}/Trp53^{KO}/FoxA1^{KO}/FoxA2^{KO}$, KP: $Kras^{CA}/Trp53^{KO}$) . **(B)** Prevalence of each single cell type in KP genotype lung specimens (normal or tumor-containing) sampled at different time (weeks after transgene induction).

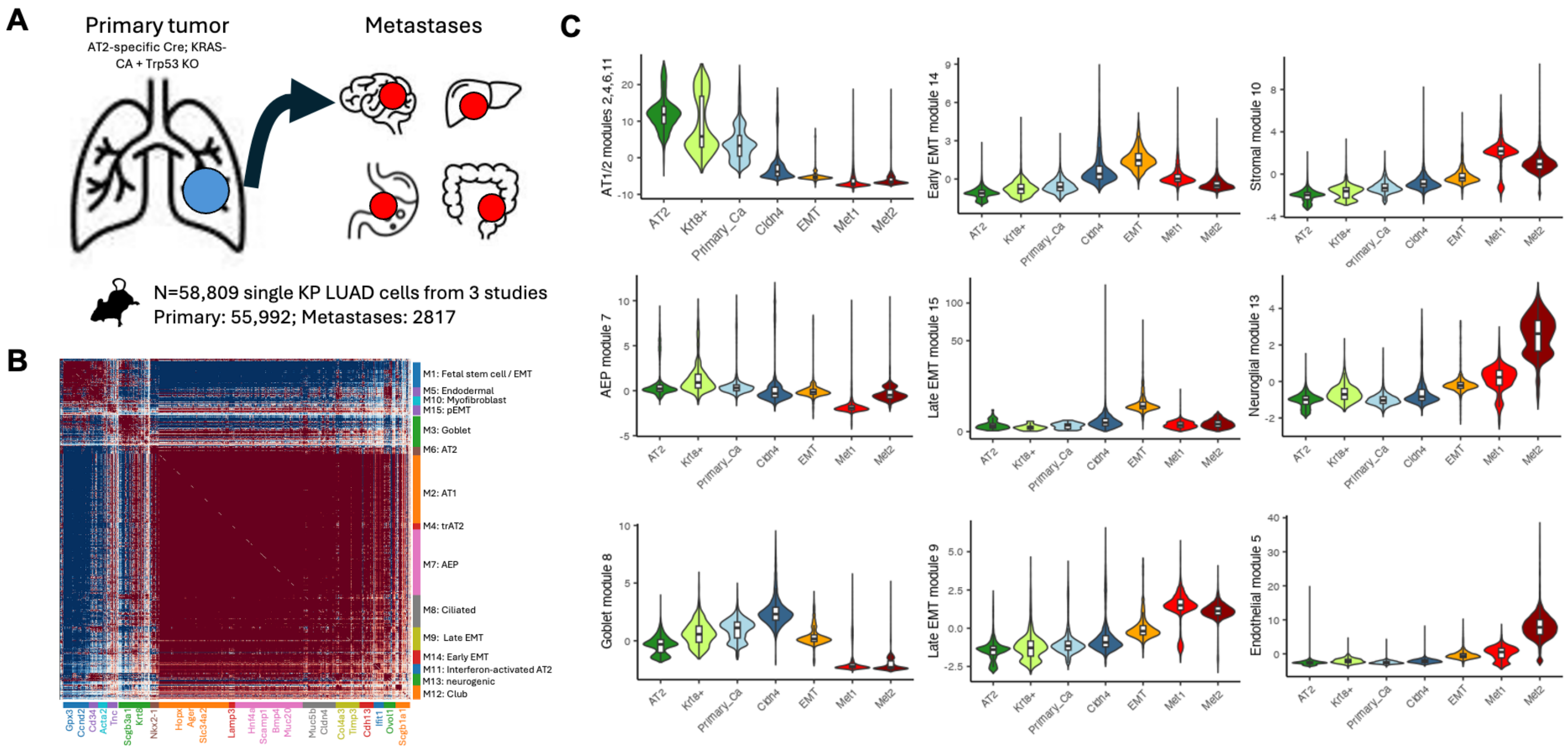

Fig. S5. Gene expression programs in the mouse LUAD scRNA datasets.
(A) Sample schematics. Single cancer cells from the primary and metastatic tumors from the KP mouse model were used. **(B)** Hotspot (*3*) identified gene transcription modules in single cancer cells. Modules were manually annotated (right). Key genes in the module were highlighted (bottom). **(C)** Expression level of each gene module in single cell types.

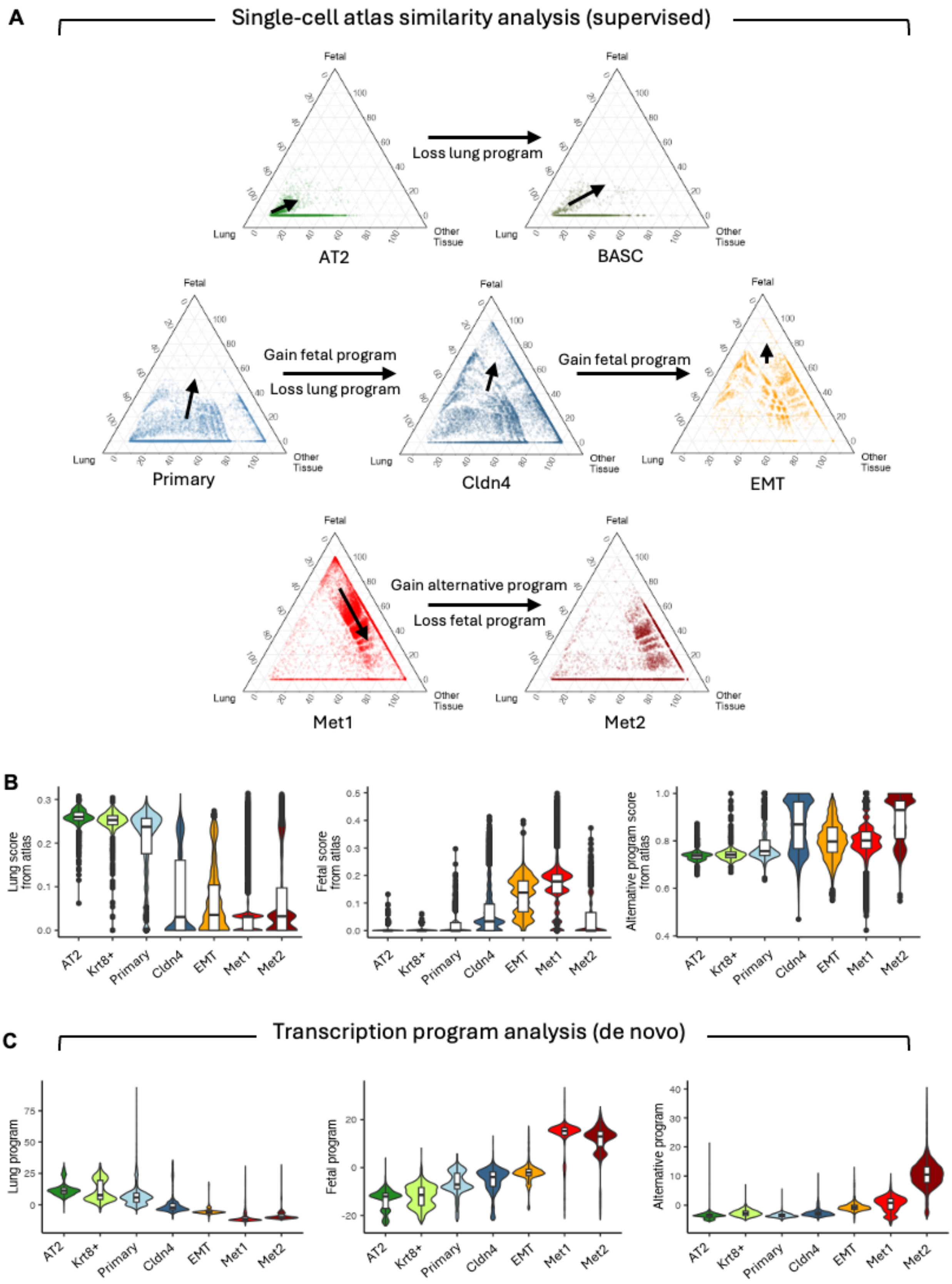
A
Single-cell atlas similarity analysis (supervised)
Fetal
Lung
Other Tissue
AT2
BASC
Loss lung program
Primary
Cldn4
EMT
Gain fetal program
Loss lung program
Gain fetal program
Met1
Met2
Gain alternative program
Loss fetal program
B
Lung score from atlas
Fetal score from atlas
Alternative program score from atlas
AT2
Krt8+
Primary
Cldn4
EMT
Met1
Met2
C
Transcription program analysis (de novo)
Lung program
Fetal program
Alternative program

Fig. S6. Expression of lung, fetal, and alternative lineage transcription programs during mouse LUAD evolution.

(A) Ternary plot of cosine similarity towards lung cells (bottom edge 0-100 from left to right), fetal cells (upper-left edge 0-100 from top to bottom), and other (alternative) cells (upper-right edge 0-100 from bottom to top) for each single cell (dots) of different types. A vector pointing from the gravitational mean of ancestor (prior step in evolution) to the one of current cell type are shown on the map (arrow). For AT2 cells we assume that 100% lung, 0% fetal and 0% others are the original state. Cosine similarity is performed with single cell RNA expression profile against normal cell reference panel (*4*). **(B)** Lung, fetal, and alternative cosine similarity of each single cell type. **(C)** Expression of *de novo* inferred (by Hotspot) lung-, fetal-, and alternative lineage programs of each single cell type.

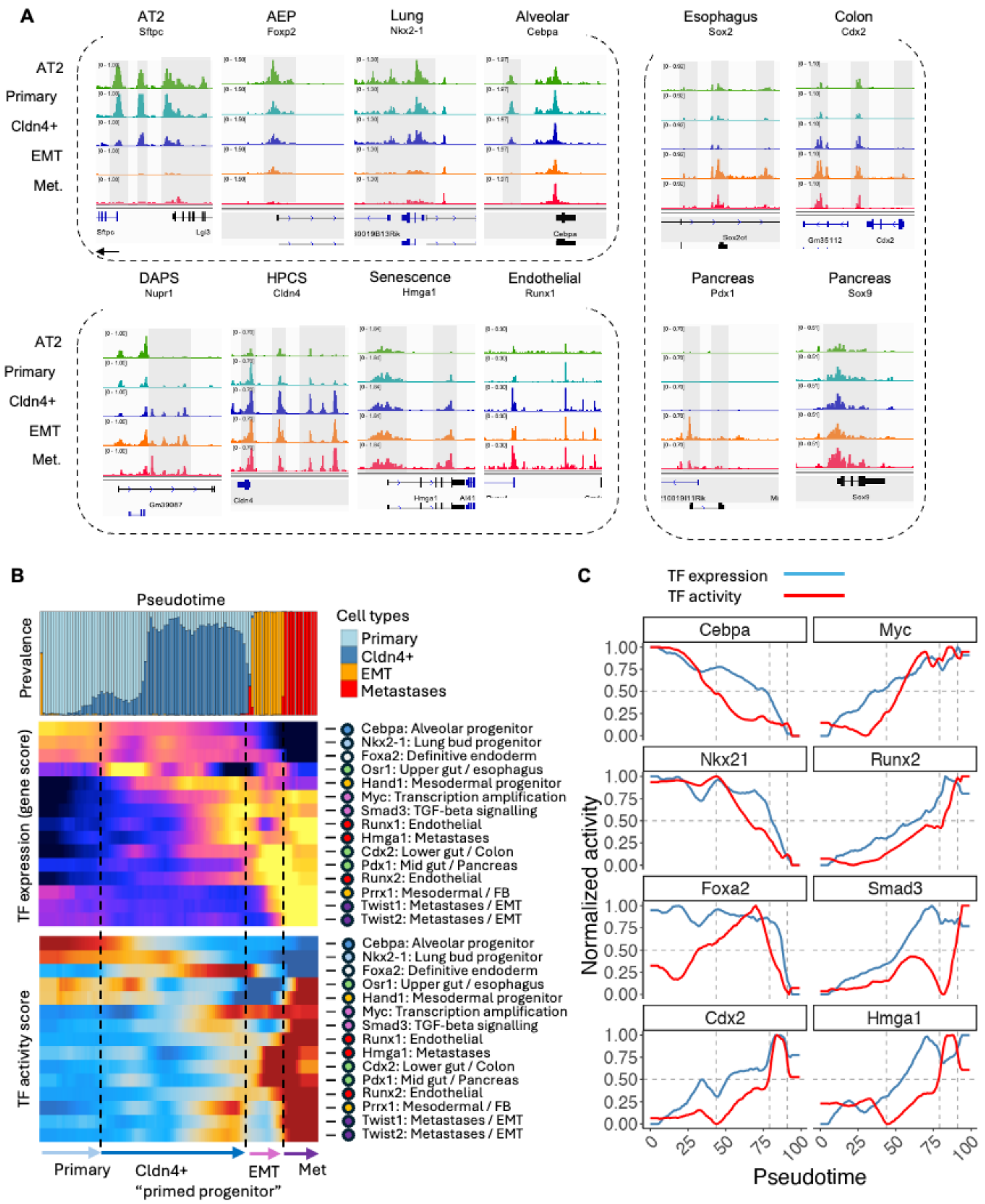

Fig. S7. Epigenomic reprogramming primes tumor trans-differentiation in mouse LUAD. **(A)** Chromatin accessibility of scATAC cell types on differentially accessible genomic regions around canonical marker genes (left) and alternative-lineage-specific transcription factors (right). **(B)** Top: the relative prevalence of each single cell type along the scATAC-inferred pseudotime

(x-axis); middle: Transcription factor expression inferred by scATAC along the pseudotime; bottom: transcription factor activity inferred by scATAC along the pseudotime. Transcription factors with time-dependent expression and activity are shown. **(C)** Normalized expression (blue) and activity (red) for key transcription factors along the pseudotime.

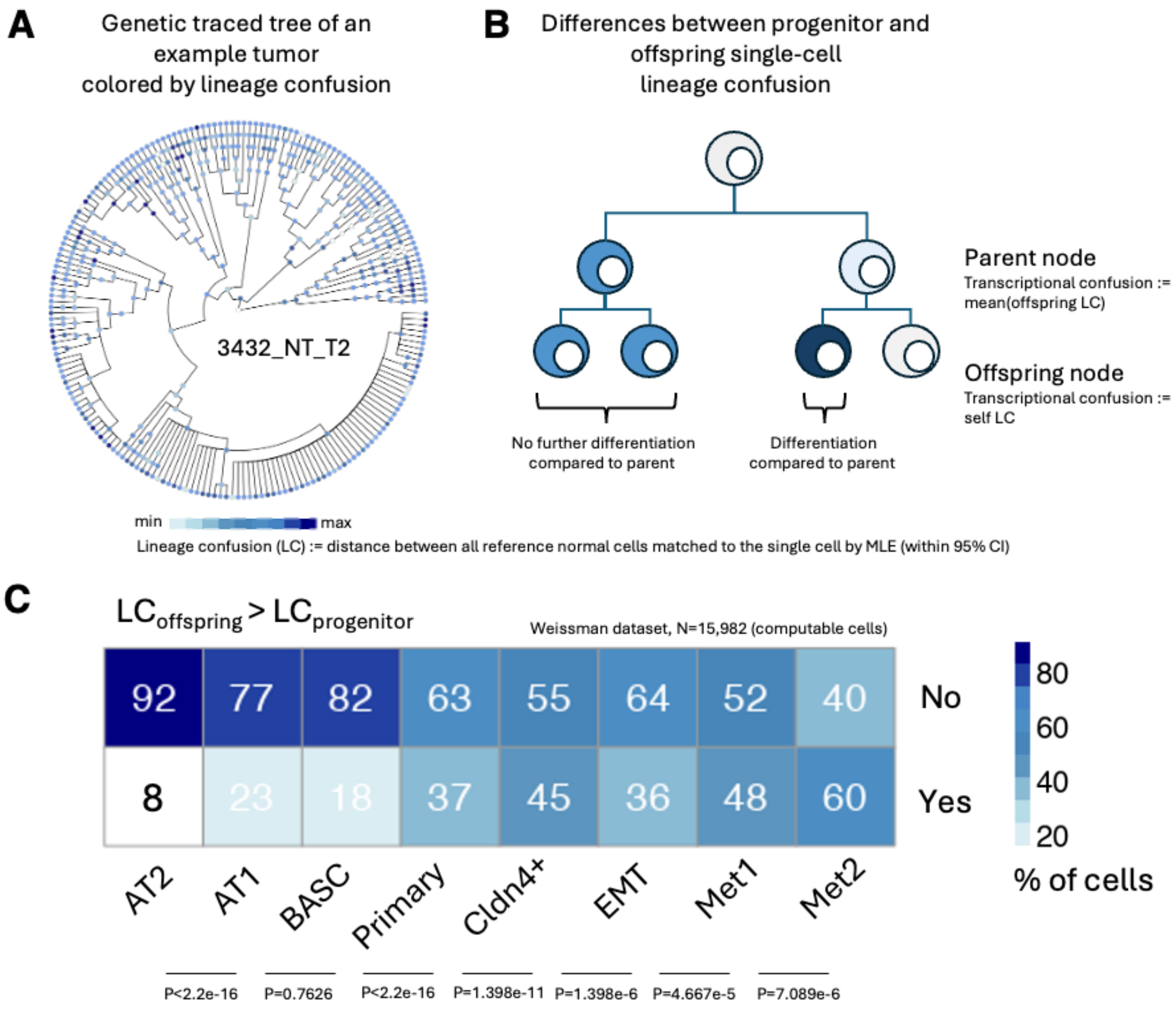

Fig. S8. Trans-differentiation of metastatic cancer cells relative to their genetic progenitor. Single cells were assigned to the phylogenetic tree by CRISPR-Cas9 DNA barcode tracing results, and annotated according to RNA expression profile. Lineage confusion (LC) of single cells is defined as the transcriptional distance between its most probable (>95%) matched reference cell types by MLE. LC of any progenitor node in the phylogenetic tree is calculated as the mean LC of all its offsprings. An increased LC in offspring relative to its progenitor node suggests trans-differentiation. **(A)** Genetic phylogenetic tree from an example tumor sample (3432_NT_T2), cells (tips) are colored by their LC. **(B)** Schematic showing how to infer the difference of LC between progenitor node and offspring cells. **(C)** Percentage of cells showing increase (or no increase) of LC for each single cell type. P-value: Fisher's exact test between groups.

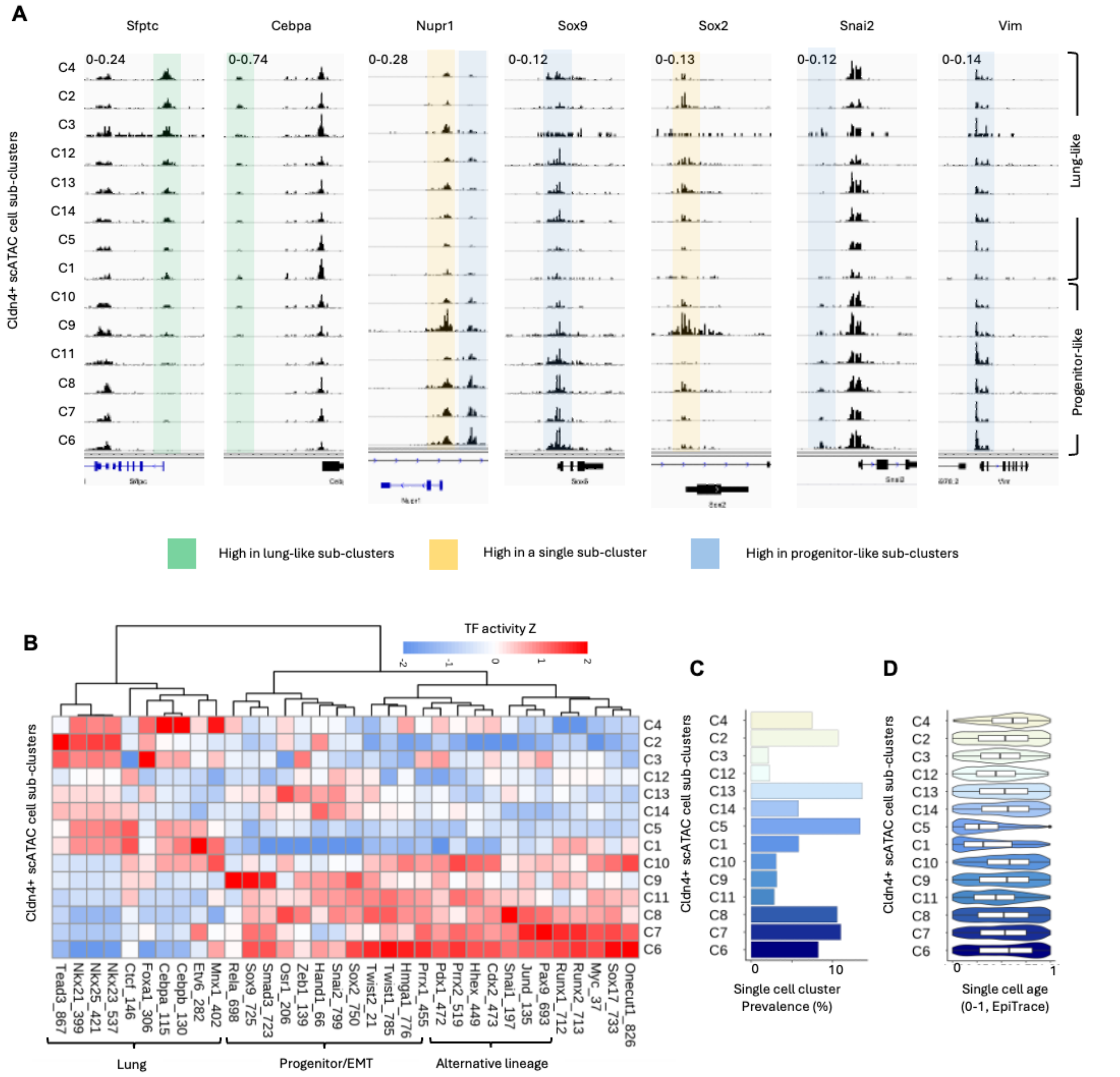

Fig. S9. Stochastic evolution of Cldn4^{+} cells prior to trans-differentiation.
(A) Chromatin accessibility of scATAC cell types on differentially accessible genomic regions around canonical marker genes for each Cldn4^{+} subcluster. The subclusters are labeled as lung-like or progenitor-like based on chromatin accessibility around lung-specific genes (*Sftpc*, *Cebpa*), progenitor cell-specific genes (*Nupr1*, *Sox9*, *Sox2*), and EMT genes (*Snai2*, *Vim*). **(B)** Transcription factor activity (scaled) of lung-specific, progenitor-or-EMT-specific, and alternative-lineage-specific transcription factors, as inferred by scATAC data, for the Cldn4^{+} subclusters. **(D)** Single cell subcluster prevalence in the Cldn4^{+} scATAC dataset. **(D)** Single cell replicational age inferred by EpiTrace for Cldn4^{+} subclusters.

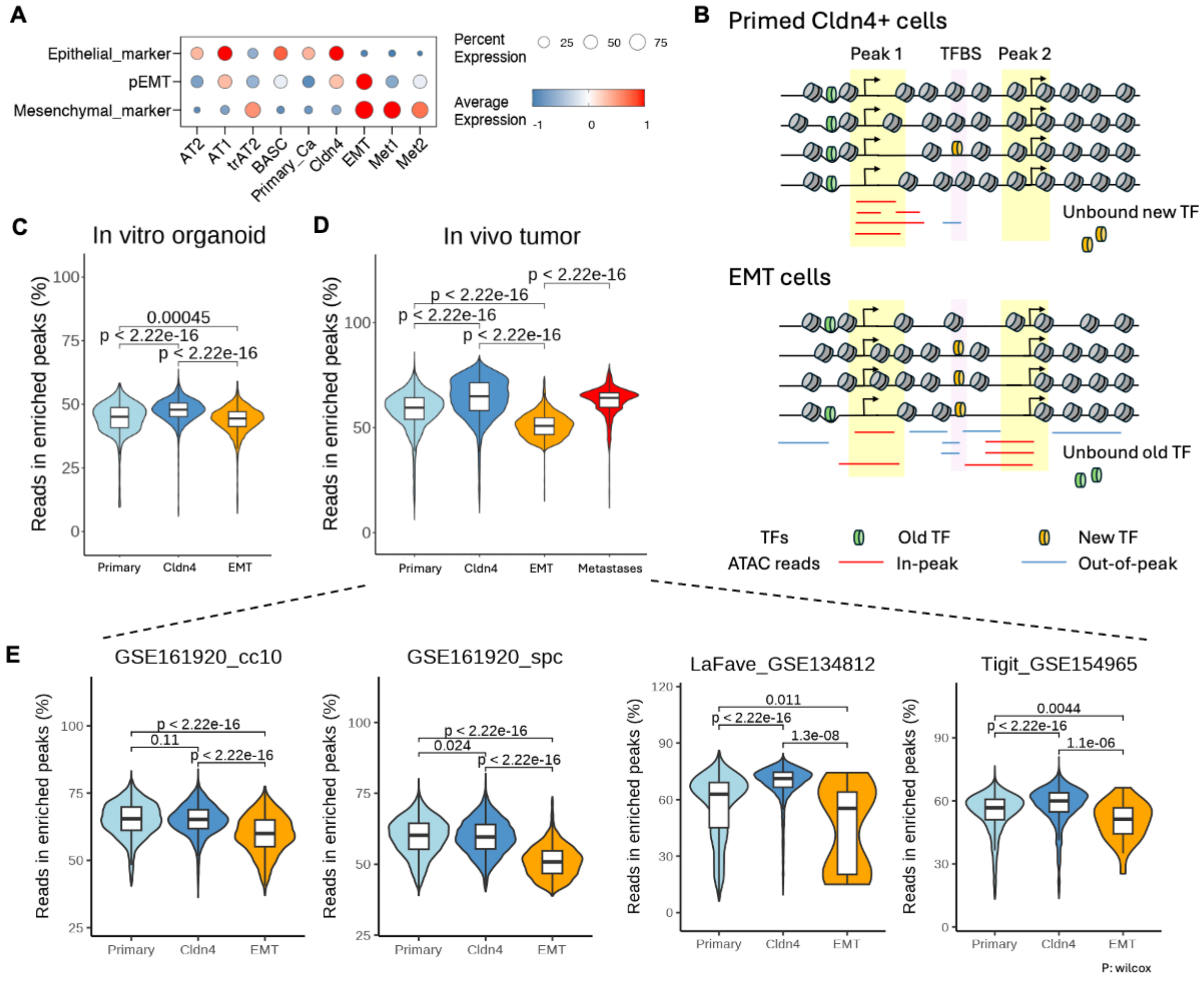

Fig. S10. Alternative lineage programs are fully activated in EMT by global chromatin accessibility increase.

(A) Gene expression score of epithelial, mesenchymal, and partial-EMT marker gene sets in each single cell type for the scATAC dataset. **(B)** Schematic of inferring global chromatin accessibility by using the fragment-ratio-in-peak parameter. Canonical peaks called by chromatin accessibility are usually promoters or enhancers (yellow), whilst regions usually inaccessible to ATAC assay in most cells are usually enhancers temporally accessible (purple) during development or are persistent heterochromatin. Measuring the ratio of fragment number in canonical ATAC peak against all fragments thus indicates how "loose" the genome is. Transiently opened chromatin region may enable new transcription factor binding to drive non-canonical lineage gene program expression. **(C)** FRIP in primary, Cldn4^{+} and EMT cancer cells in the *in vitro* LUAD organoid. **(D)** FRIP in *in vivo* LUAD tumor cells. **(E)** FRIP in *in vivo* tumor cells, split by dataset.

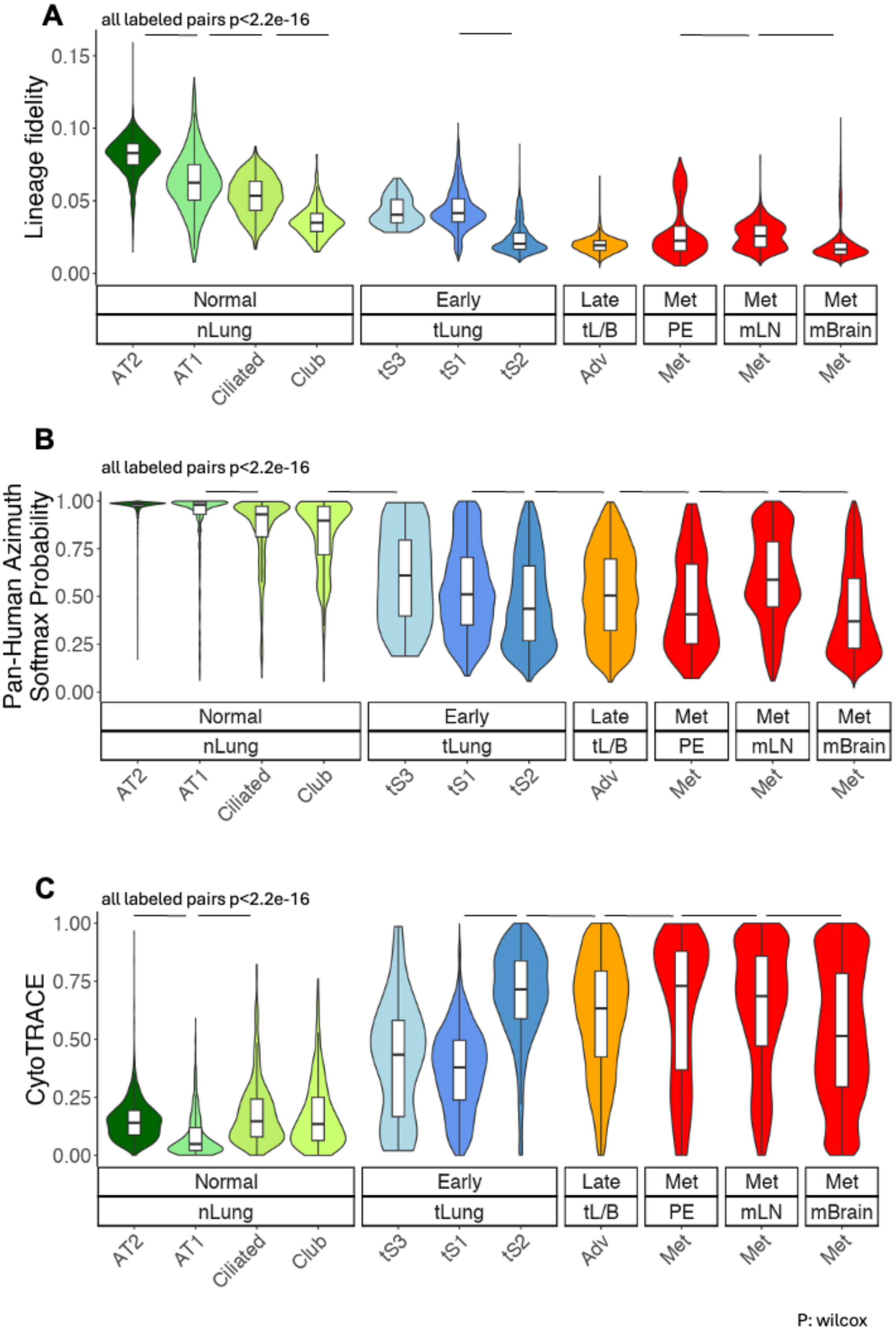
A
all labeled pairs p<2.2e-16
Lineage fidelity
0.15
0.10
0.05
0.00
Normal
nLung
Early
tLung
Late
tL/B
Met
PE
Met
mLN
Met
mBrain
AT2
AT1
Ciliated
Club
tS3
tS1
tS2
Adv
Met
Met
Met
B
all labeled pairs p<2.2e-16
Pan-Human Azimuth Softmax Probability
1.00
0.75
0.50
0.25
0.00
Normal
nLung
Early
tLung
Late
tL/B
Met
PE
Met
mLN
Met
mBrain
AT2
AT1
Ciliated
Club
tS3
tS1
tS2
Adv
Met
Met
Met
C
all labeled pairs p<2.2e-16
CytoTRACE
1.00
0.75
0.50
0.25
0.00
Normal
nLung
Early
tLung
Late
tL/B
Met
PE
Met
mLN
Met
mBrain
AT2
AT1
Ciliated
Club
tS3
tS1
tS2
Adv
Met
Met
Met
P: wilcox

Fig. S11. Stemness and lineage fidelity in human lung cancer.
(A) Lineage fidelity of human LUAD cancer cells. **(B)** Probability of correct matching to a normal reference cell of the same cells. **(C)** Stemness score (CytoTRACE) of the same cells.

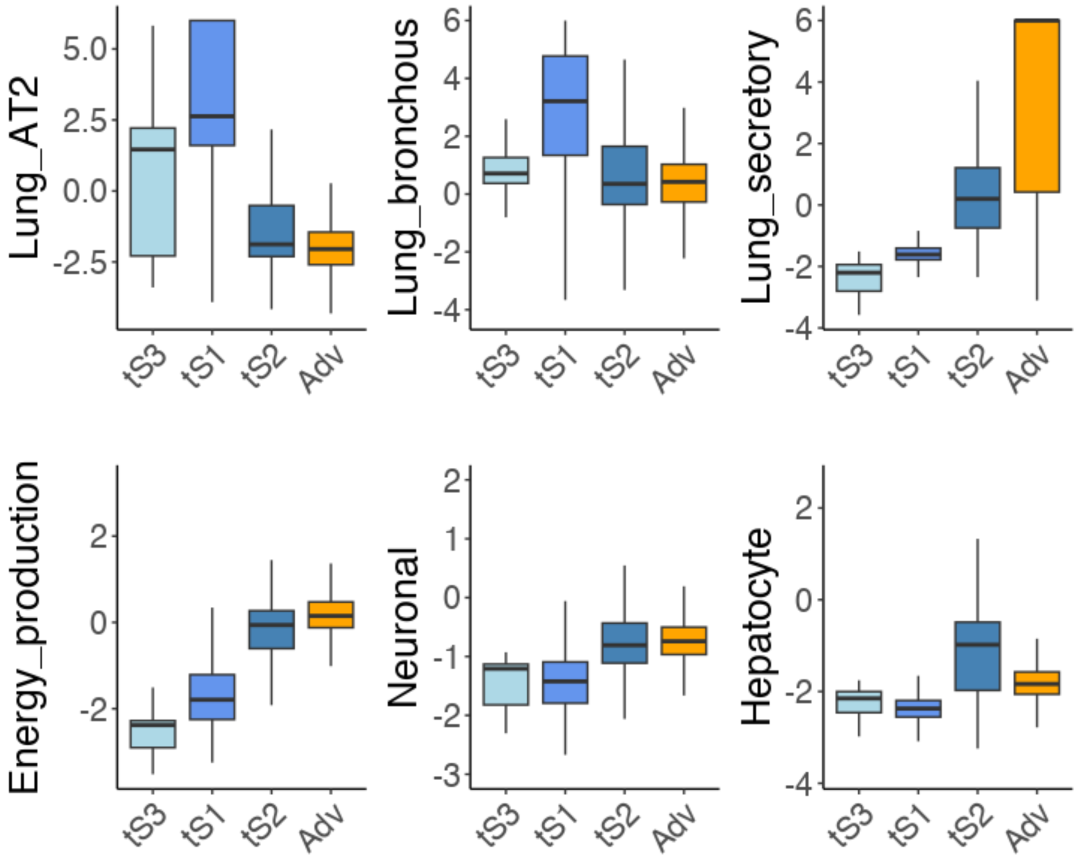

Fig. S12. Gene module expression in primary human lung cancer cell types.
Expression of gene modules inferred by Hotspot were shown for the early (tS3, tS1, tS2) and advanced-stage (Adv) tumor cells.

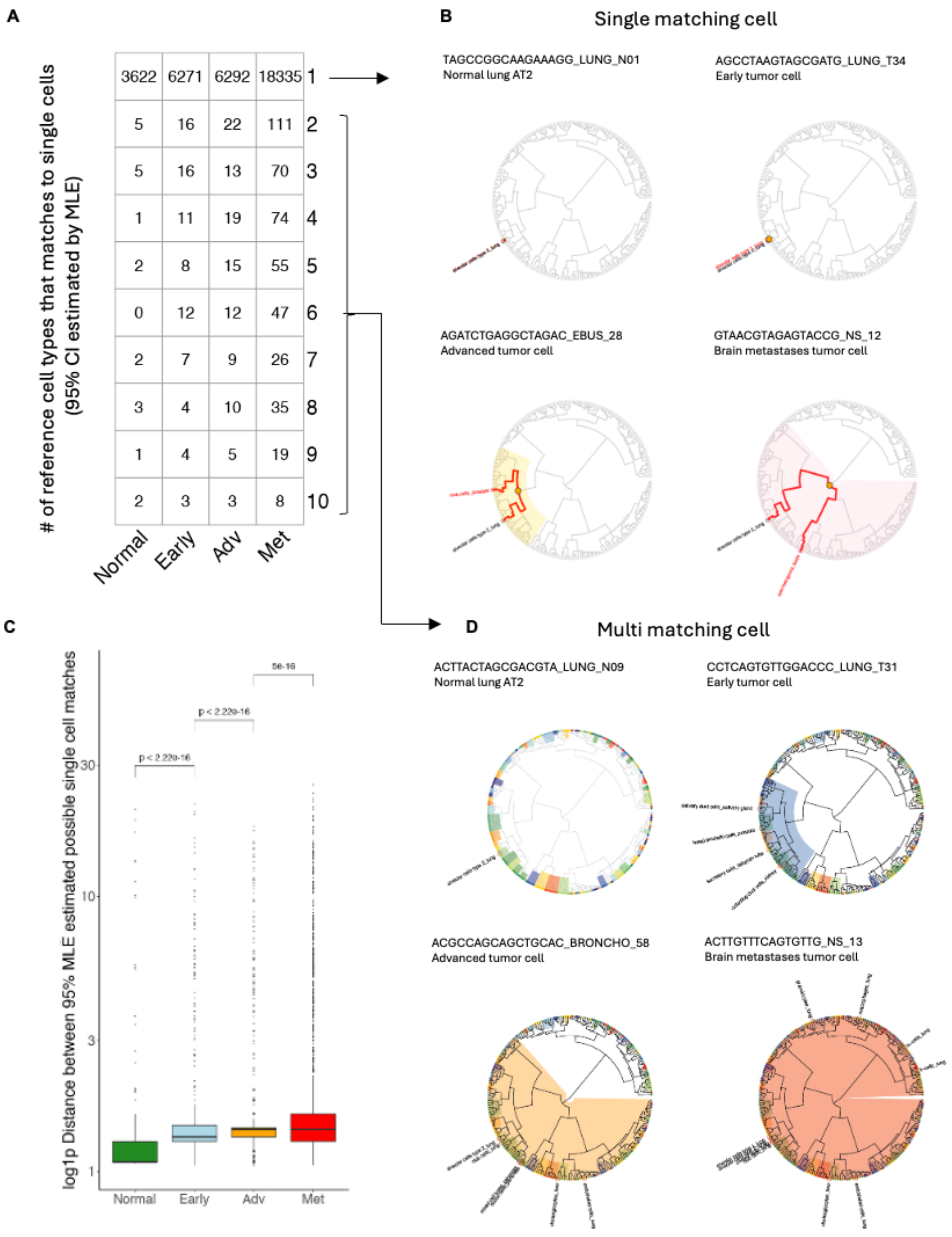

Fig. S13. Transformation of lineage in single human tumor cells.

(A) Table of the number of single cells of a specific class (columns) that matched to a given number of normal reference cell type (rows). **(B)** Examples of traverse trajectory from the normal AT2 cell to the matched reference cell on the transcription similarity tree. The transcriptional distance between observed state and normal AT2 cells gradually increase from early to advanced and metastases tumor cells. **(C)** Transcriptional distance between normal AT2 to observed state for each cell class. **(D)** Examples of normal reference cell match the rare multi-matched cells. Large colored shade highlights the putative branch covered by their most-recent common ancestor (MRCA). The MRCA covers gradually larger transcriptional space as the tumor evolves.

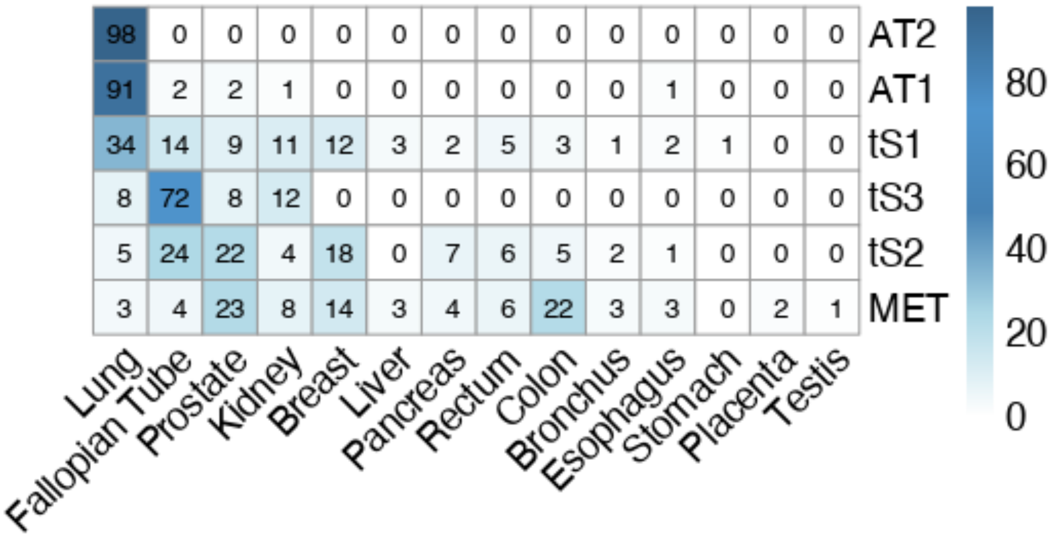

Fig. S14. Tumor cell progressively switches to developmentally distant lineages.
Table of the percentage from each single cell types (rows) that matched to normal reference cells from specific lineage (columns).

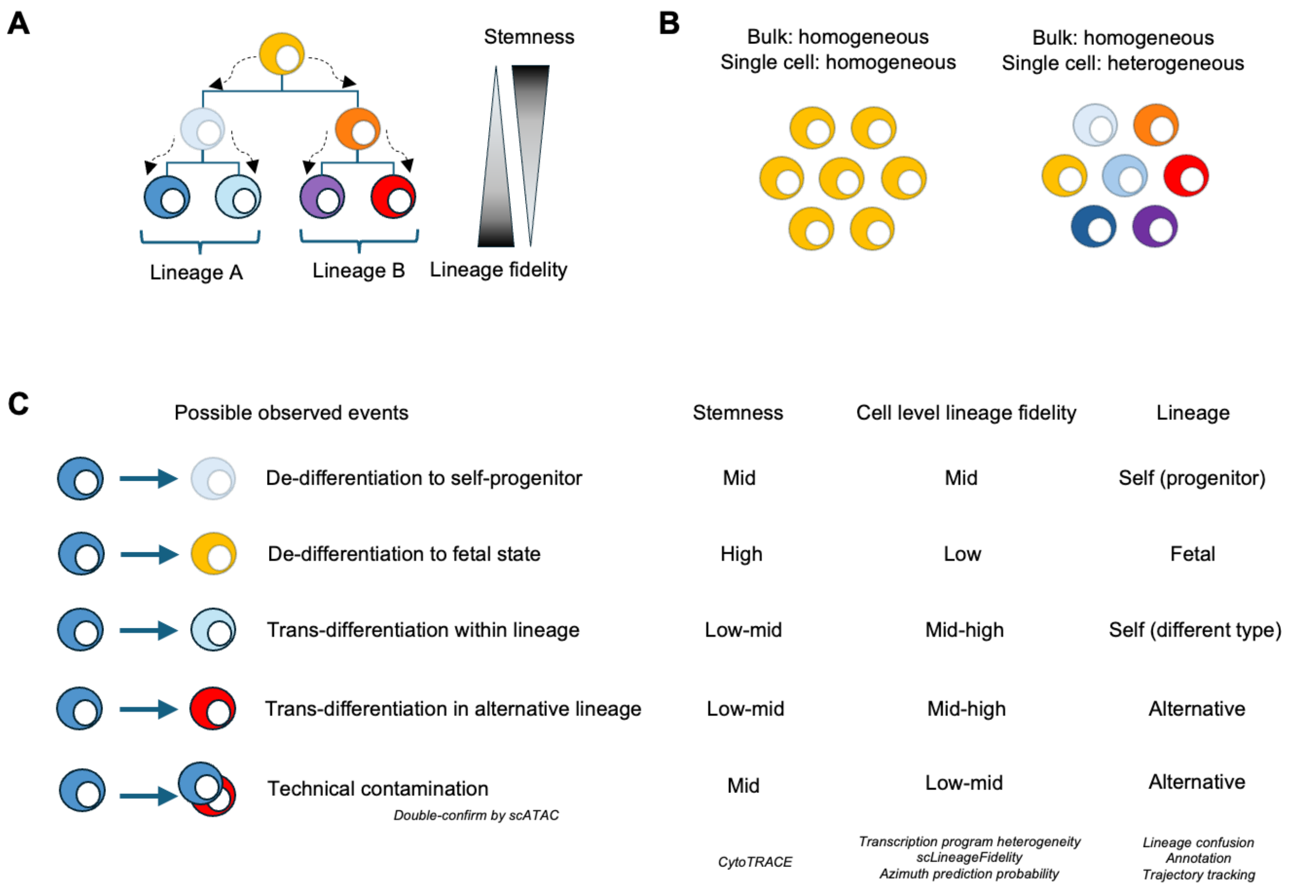

Fig. S15. Classification of transformation events.
(A) A normal developmental tree of cells. Stemness is higher in the progenitor and lower in the terminally differentiated progenies. Conversely, lineage fidelity is high in the progenies and low in the progenitor. **(B)** Transcriptional heterogeneity in single-cell level is not always reflected on the population (bulk) level. **(C)** Classes of possible events, and their corresponding stemness, lineage fidelity, and lineage annotation biomarkers. Methods to detect these biomarkers are shown in italic.

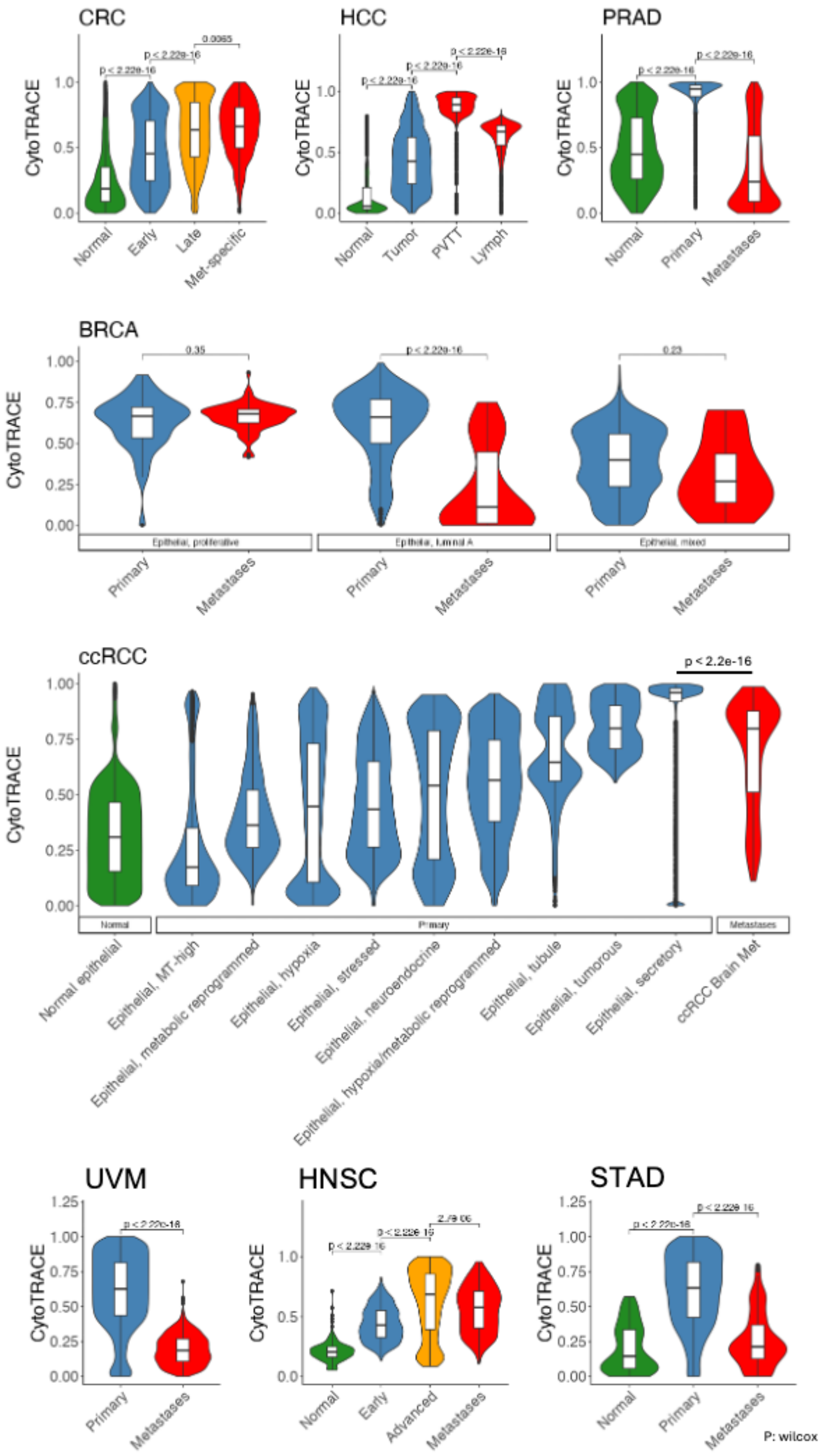
CRC
HCC
PRAD
CytoTRACE
p < 2.22e-16
0.0065
Normal
Early
Late
Met-specific
Tumor
PVTT
Lymph
Primary
Metastases
BRCA
0.35
0.23
Epithelial, proliferative
Epithelial, mixed
ccRCC
p < 2.2e-16
Normal
Primary
Metastases
Normal epithelial
Epithelial, MT-high
Epithelial, metabolic reprogrammed
Epithelial, hypoxia
Epithelial, stressed
Epithelial, neuroendocrine
Epithelial, hypoxia/metabolic reprogrammed
Epithelial, tubule
Epithelial, tumorous
Epithelial, secretory
ccRCC Brain Met
UVM
HNSC
STAD
Advanced
P: wilcox

Fig. S16. Stemness of normal, primary, and metastatic tumor cells in other human cancers. Non-lung cancer CytoTRACE scores are shown. Cells used are similar to the cells shown in Figure 3. Classification of ccRCC primary cells is according to the original publication annotation.

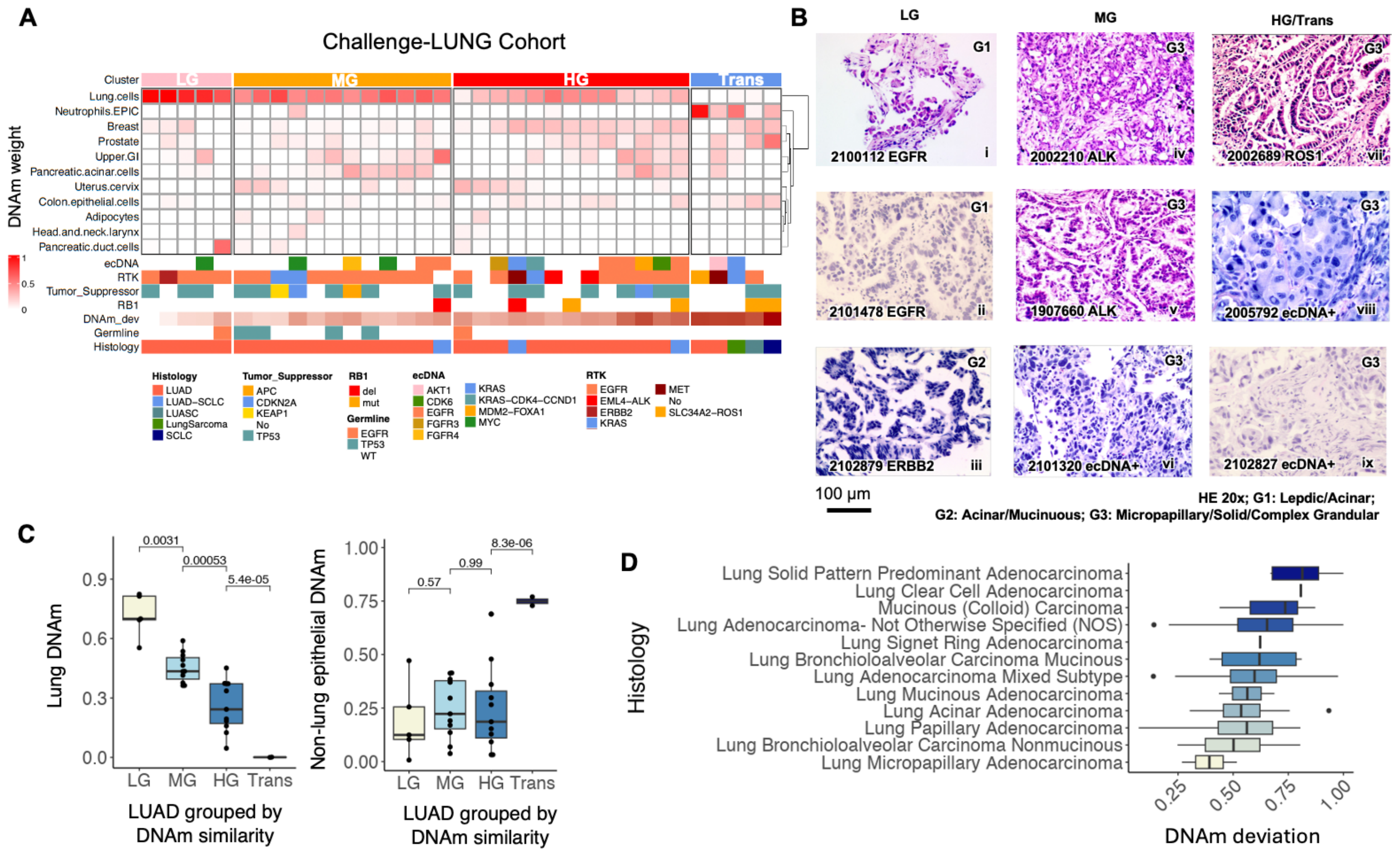

Fig. S17. Establishment of DNAm-based lineage trans-differentiation index.
(A) NNLS-decomposed LUAD primary tumor DNAm profile as a mixture of normal reference tissue DNAm profile. Weights in the mixture are shown on the heatmap. Sample-specific meta-data are shown at the bottom of the heatmap. Tumors are classified by hierarchical clustering of this deconvolution result to low-grade (LG), mid-grade (MG), high-grade (HG) and trans-differentiated (Trans)

classes. **(B)** H&E staining of the sampled tumors. **(C)** Lung- and non-lung epithelial DNAm for the four classes of tumor. **(D)** DNAmDev index from the TCGA LUAD cohort, grouped by their histopathological status.

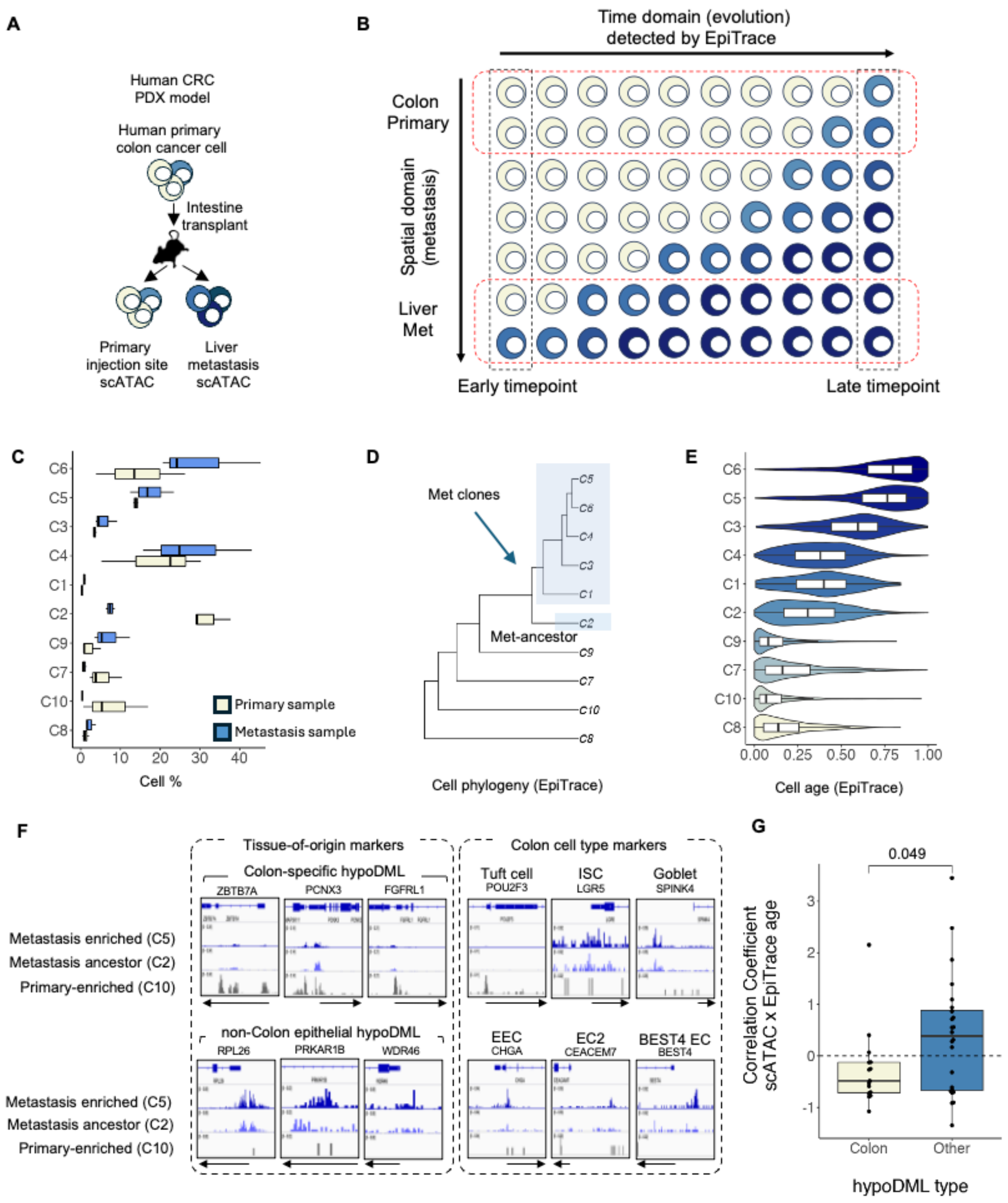

Fig. S18. Single cell age estimation tracks the emergence of trans-differentiated metastases from the primed progenitor in human CRC PDX model.

(A) Schematic of the experiment. Human primary colorectal cancer (CRC) was transplanted to mouse ileum, and the tumor formed from intestine (primary site) and liver (metastases) are subjected to scATAC sequencing. N=3 for each site. **(B)** Schematic of analysis. Spatial

dissemination of tumor cells are tracked by the relative prevalence of single cell clusters in different sample sites, and the temporal evolution of tumor cells are tracked by estimating cell replicational age. **(C)** Prevalence of each single cell cluster in primary (white) and metastases (blue) samples. **(D)** EpiTrace-built single cell cluster phylogenetic tree, highlighting the metastases-enriched clones (C1/3/4/5/6) and their putative ancestor (C2). **(E)** Cell replicational age (by EpiTrace) of each single cell cluster. **(F)** Chromatin accessibility on colon- and non-colon specific enhancers, and cell-type-specific regions. The primary-site-enriched cells are epigenetically similar to colon tuft cells. The tuft-specific epigenome is gradually lost during the transition towards metastases. Metastases-enriched cells show not only other colon cell type-specific epigenetic profiles but also non-colon features. **(G)** Non-colon-specific peaks show significantly higher correlation to single cell replicational age, suggesting that they are gradually opened during tumor progression. P-value: Wilcox.

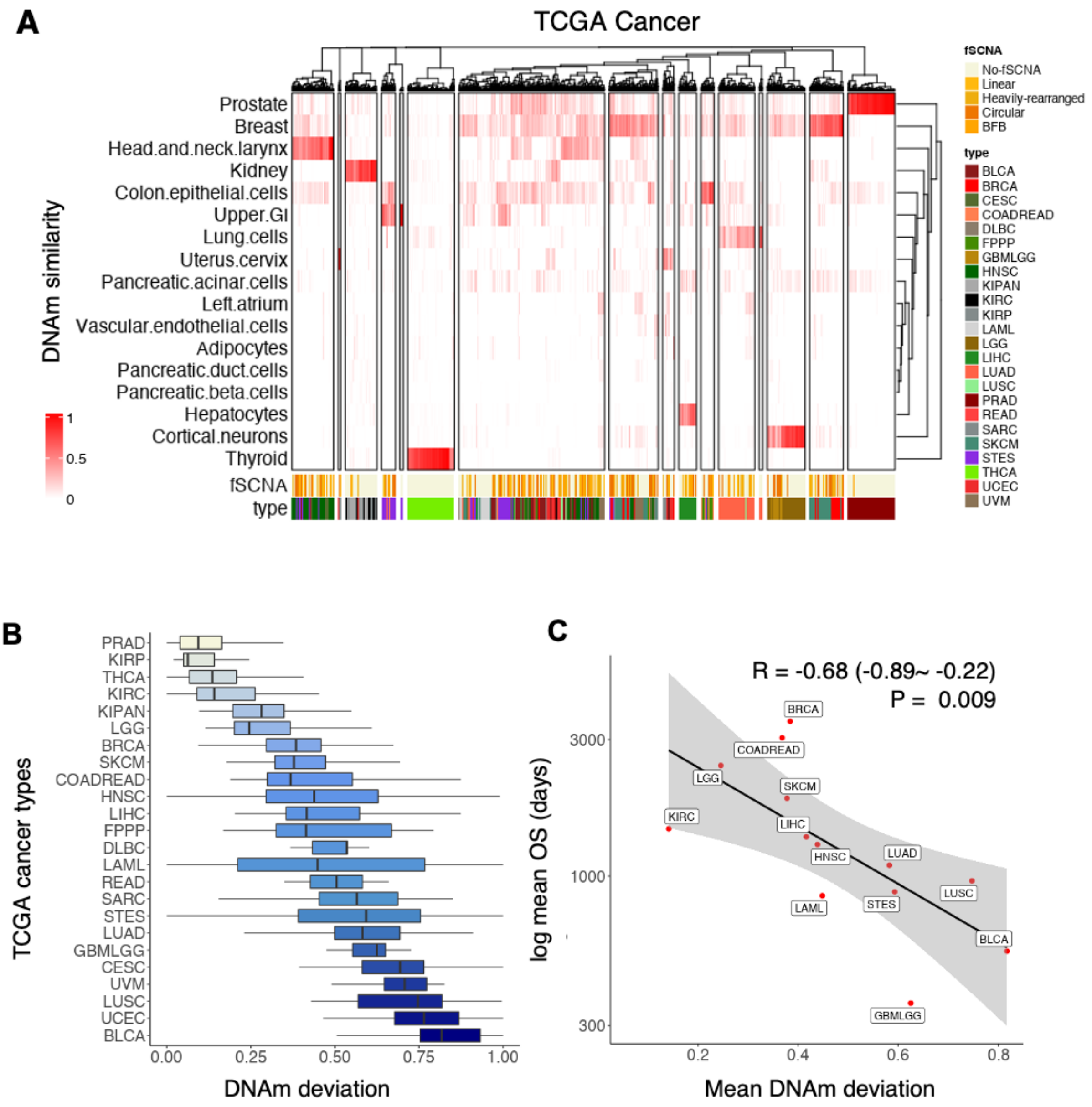

Fig. S19. Trans-differentiation across human cancer in TCGA dataset.
(A) NNLS-decomposed DNAm profile as a mixture of normal reference tissue DNAm profile. Weights in the mixture are shown on the heatmap. Sample-specific meta-data (cancer type and focal short copy number amplification, fSCNA) are shown at the bottom. **(B)** DNAmDev index for each tumor type. For each tumor type, we infer the "original type" by vote-of-majority principle, as we expect most tumors still show similarity to their original tissue type. **(C)** Mean DNAmDev index and overall survival (OS) of each tumor type. 13 types of tumors have sufficient data for analysis.

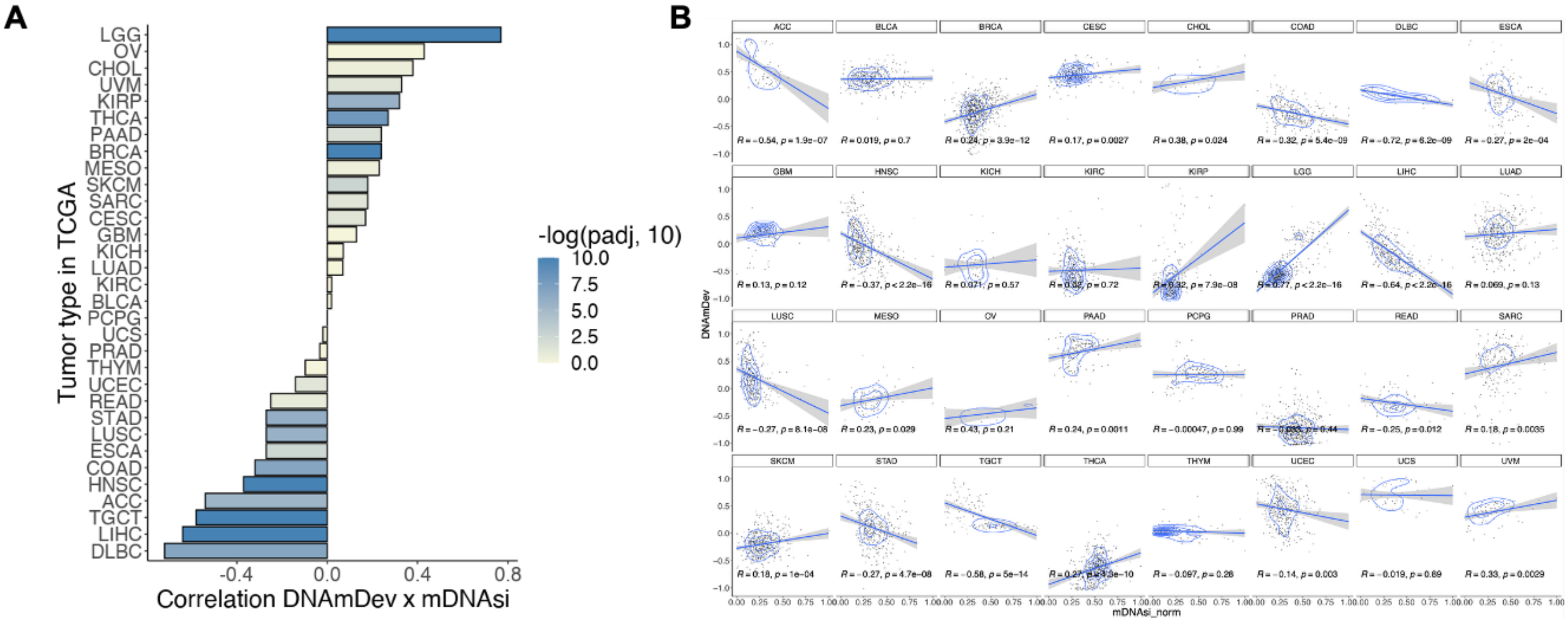

Fig. S20. Comparing lineage trans-differentiation index (DNAmDev) and fetal-like index (mDNAsi).
(A) Overall correlation between mDNAsi and DNAmDev for each tumor type in the TCGA cohort. P-value for correlation is BH adjusted. **(B)** Scatter plot of DNAmDev and mDNAsi for each tumor sample in the cohort. P-value and R: Pearson's.

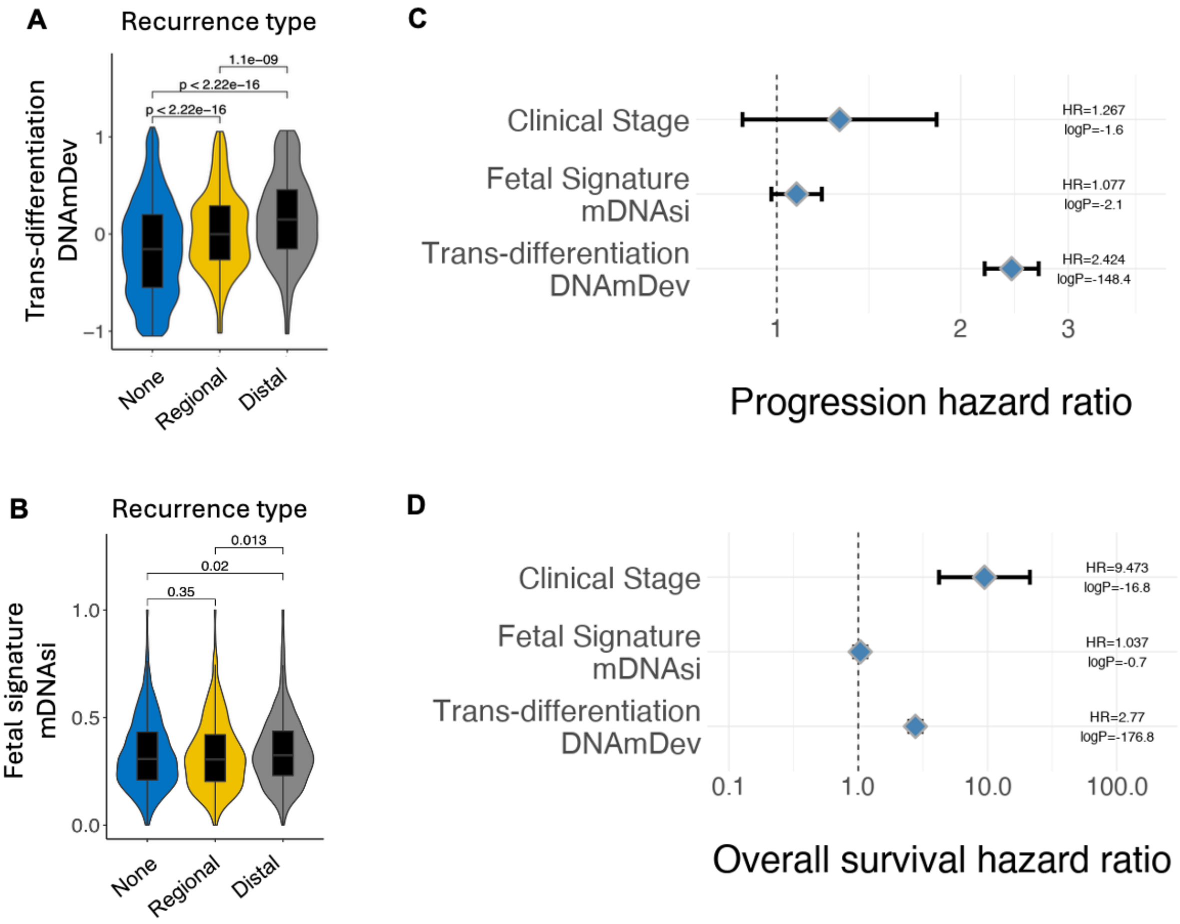

Fig. S21. Trans-differentiation promotes clinical progression.
(A) DNAmDev index for tumors that not progressed (none), progressed regionally, or metastasized distally. **(B)** mDNAsi index for tumors that not progressed (none), progressed regionally, or metastasized distally. **(C)** Forest plot of hazard ratio for progression under Cox model using clinical stage, mDNAsi, and DNAmDev as parameters. **(D)** Forest plot of hazard ratio for overall survival under Cox model using clinical stage, mDNAsi, and DNAmDev as parameters.

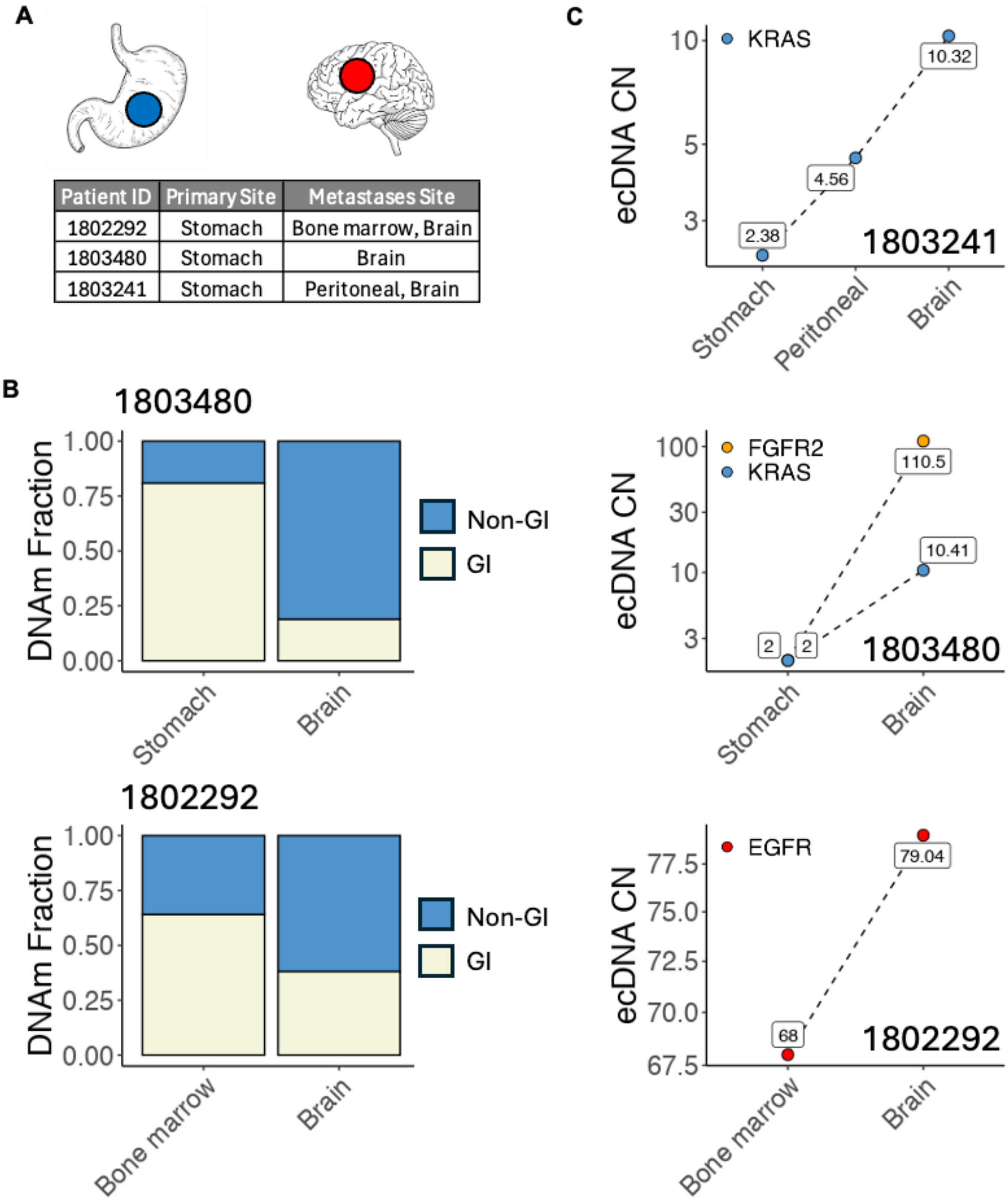

Patient ID	Primary Site	Metastases Site
1802292	Stomach	Bone marrow, Brain
1803480	Stomach	Brain
1803241	Stomach	Peritoneal, Brain

Fig. S22. Genetic gain of MAPK signaling associates with lineage trans-differentiation and metastases.
(A) Clinical features of the three STAD brain metastases patients. **(B)** NNLS-decomposed DNAm weight from gastrointestinal tract (GI, original) or non-gastrointestinal (non-GI, alternative) tissues for two patients with available samples undergone paired primary-metastases DNAm sequencing. **(C)** MAPK pathway oncogene ecDNA amplification copy number in primary and metastases samples for each patient. The relative copy number for each ecDNA is shown.

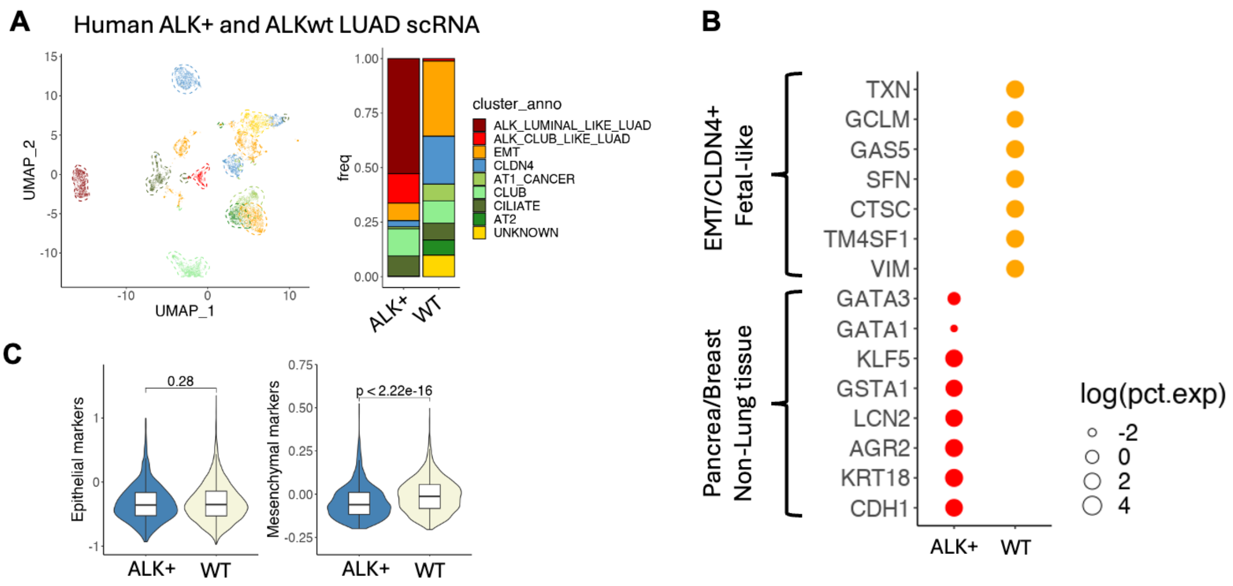

Fig. S23. Genetic gain of MAPK signaling associates with trans-differentiation in human LUAD scRNA.

(A) UMAP of human ALK-fusion (ALK^{+}) and ALK-wildtype (ALK^{wt}) scRNA cells. **(B)** Epithelial and mesenchymal gene module scores of ALK^{+} and ALK^{wt} cells. **(C)** Differential gene expression between ALK^{+} and ALK^{wt} cells related to lineage trans-differentiation.

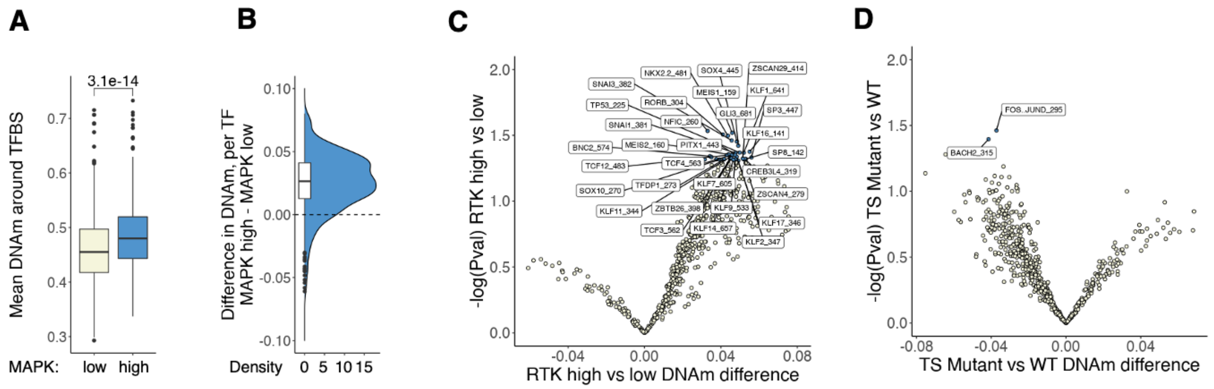

Fig. S24. Genetic gain of MAPK signaling associates with hypermethylation of EMT TFBS. **(A)** Mean DNAm value (beta) in +/- 200 bp around all putative TFBS (defined by cisbp) in MAPK-low and MAPK-high tumor samples from the Challenge-Lung cohort. **(B)** Boxplot of difference of DNAm value around TFBS between MAPK-high and low tumors, for each TF. Most TF are hypermethylated in MAPK-high samples. **(C)** Volcano plot of differential DNA methylation around TFBS for each TF between MAPK-low and MAPK-high tumors. TFBS class with significant DNAm changes are highlighted. **(D)** Volcano plot of differential DNA methylation around TFBS for each TF between tumor-suppressor-mutated and tumor-suppressor-wildtype tumors. TFBS class with significant DNAm changes are highlighted.

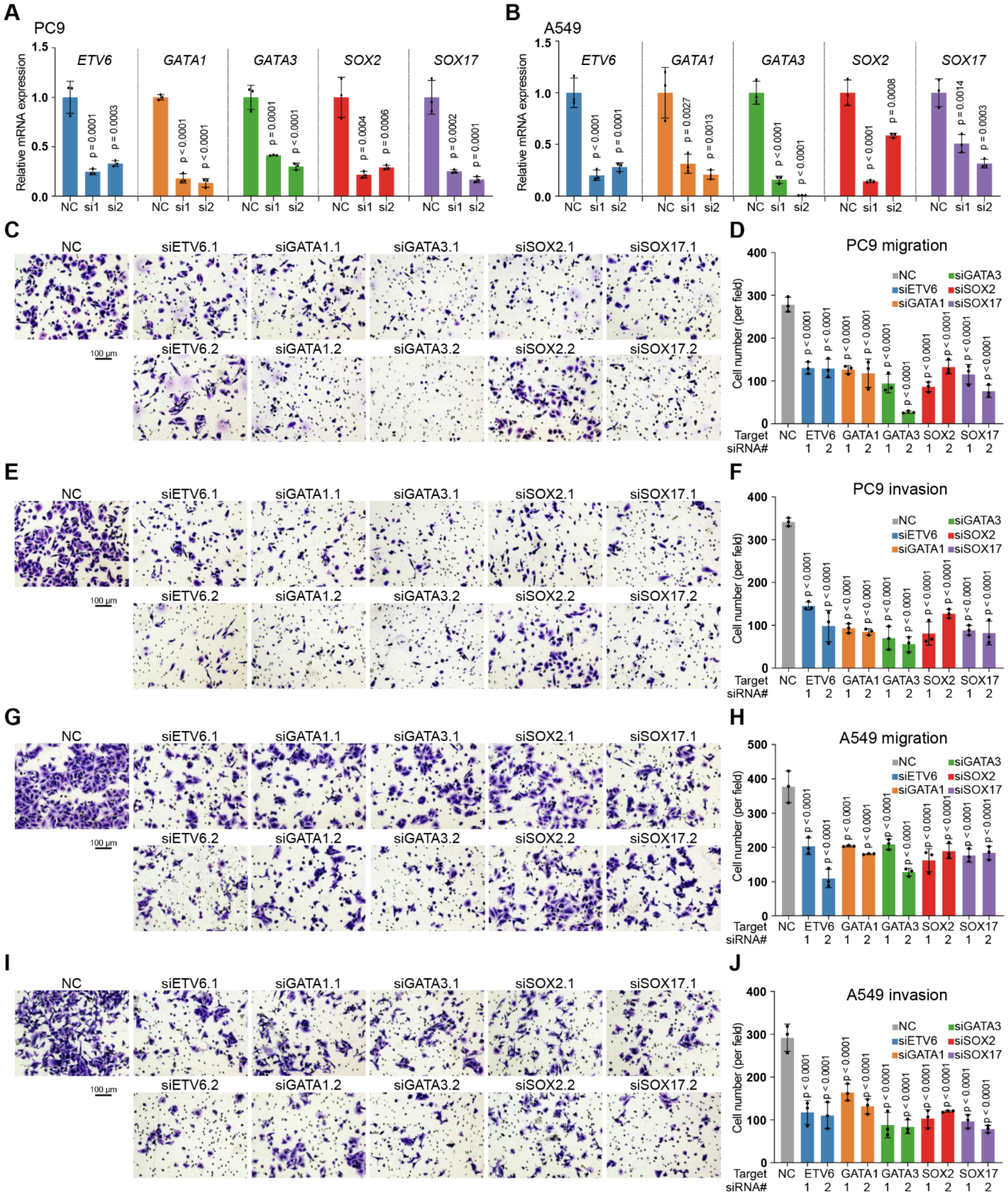

Fig. S25. Knockdown of alternative-lineage transcription factors impairs migration and invasion of lung cancer cells.

(A-B) qRT-PCR analysis of the knockdown efficiency of five transcription factor-specific siRNAs in PC9 (A) and A549 (B) cells. **(C-D)** Representative images (C) and quantification of migrated cells (D) in transwell migration assays of PC9 cells in the indicated groups. **(E-F)** Representative

images (E) and quantification of invaded cells (F) in transwell invasion assays of PC9 cells in the indicated groups. **(G-H)** Representative images (G) and quantification of migrated cells (H) in transwell migration assays of A549 cells in the indicated groups. **(I-J)** Representative images (I) and quantification of invaded cells (J) in transwell invasion assays of A549 cells in the indicated groups. Data are presented as means ± SDs; one-way ANOVA followed by Dunnett's multiple-comparisons test was used for statistical analysis (A-B, D, F, H, J). Scale bar, 100 μm.

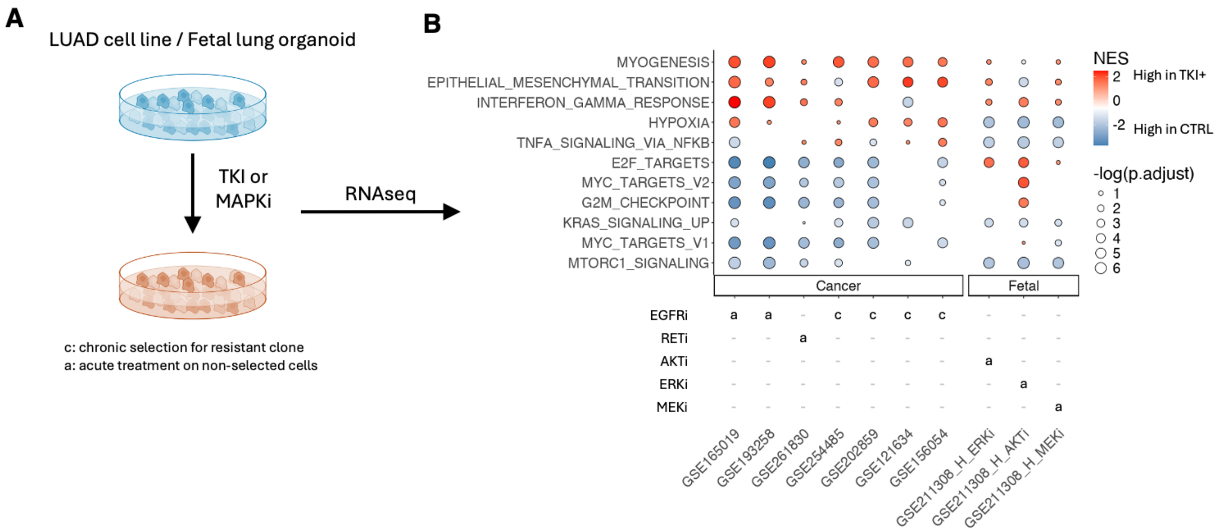

Fig. S26. TKI treatment consistently activates myogenic programs via ERK/MEK signaling. **(A)** Schematic of the experiment. Cells (or organoid) are subjected to tyrosine kinase inhibitor drug treatment before sequencing. 8 datasets sufficing these criteria are collected for this analysis. **(B)** GSEA scores (NES) of significantly enriched Hallmark pathways in any experiment. Cells under chronic TKI treatment are denoted as "c" and acute treatment is denoted by "a".

Legends for Tables S1-S6

Table S1. Total progenitor size estimated by TarCA for mouse LUAD.

For each cell type, the table records the number of phylogenetic trees containing the cell type (n_tree), the minimal Np (min), the maximal Np (max), the median Np (median), the 25% Np (q1), the 75% Np (q3), 25%-75% Np range (iqr), mean Np (mean), standard deviation of Np (sd).

Table S2. Statistical test results for genetic mutation altering DNAmDev index value.

Wilcox test results of DNAmDev index between different groups of samples carrying denoted genotype are recorded in this table.

Table S3. Datasets and reference databases used in the study.

The first sheet documents datasets used in the study, including their accession ID, relevant animal species, associated disease type, data type (single-cell RNA-seq, single-cell ATAC-seq, bulk RNA-seq, or DNAm-microarray), reference for the dataset, and the figures using these data. The second sheet documents reference datasets used in the study.

Table S4. siRNAs and primers used for transwell analysis.

The target sequences of siRNAs and primer sequences used for transwell migration and invasion assays, including raw data for the transwell analysis shown in Fig. S25.

Table S5. Software packages and their versions used in the study.

Software name/package name used in this study and the respective version number are recorded in this table.

Table S6. Gene sets used in the study.

Human and mouse gene sets used in the study (with respect to each specified analysis) are recorded in this table.

References

1. D. Yang *et al.*, Lineage tracing reveals the phylodynamics, plasticity, and paths of tumor evolution. *Cell* **185**, 1905-1923 e1925 (2022).
2. L. M. LaFave *et al.*, Epigenomic State Transitions Characterize Tumor Progression in Mouse Lung Adenocarcinoma. *Cancer Cell* **38**, 212-228 e213 (2020).
3. D. DeTomaso, N. Yosef, Hotspot identifies informative gene modules across modalities of single-cell genomics. *Cell Syst* **12**, 446-456 e449 (2021).
4. R. Wang *et al.*, Construction of a cross-species cell landscape at single-cell level. *Nucleic Acids Res* **51**, 501-516 (2023).
